# Boosting knowledge on occupational exposure to microbial contamination in Portuguese carpentries

**DOI:** 10.3389/fpubh.2025.1574881

**Published:** 2025-06-06

**Authors:** Marta Dias, Bianca Gomes, Pedro Pena, Renata Cervantes, Margarida Rodriguez, Bruna Riesenberger, Liliana Marques, Elisabete Carolino, Magdalena Twarużek, Robert Kosicki, Ewelina Soszczynska, Liliana Aranha Caetano, Susana Viegas, Carla Viegas

**Affiliations:** ^1^NOVA National School of Public Health, Public Health Research Centre, Comprehensive Health Research Center, CHRC, REAL, CCAL, NOVA University Lisbon, Lisbon, Portugal; ^2^H&TRC – Health & Technology Research Center, ESTeSL – Escola Superior de Tecnologia e Saúde, Instituto Politécnico de Lisboa, Lisboa, Portugal; ^3^CE3C—Center for Ecology, Evolution and Environmental Change, Faculdade de Ciências, Universidade de Lisboa, Lisbon, Portugal; ^4^Kazimierz Wielki University, Faculty of Biological Sciences, Department of Physiology and Toxicology, Chodkiewicza, Bydgoszcz, Poland; ^5^Research Institute for Medicines (iMed.uLisboa), Faculty of Pharmacy, University of Lisbon, Lisbon, Portugal

**Keywords:** occupational exposure, carpentry, woodworkers, wood dust, fungi

## Abstract

**Introduction:**

Wood industry workers face health risks due to exposure to microorganisms and their metabolites. This study aimed to characterize seasonal microbial contamination, antifungal resistance, mycotoxins, cytotoxicity, and particulate matter in Portuguese carpentries, to reduce exposure and promote safe working conditions.

**Methods:**

Conducted in six carpentries in Lisbon, Portugal, the sampling strategy encompassed active and passive sampling methods to assess microbial contamination. A Handheld Particle Counter HH3016-IAQ was used to monitor particulate matter size, temperature, and humidity.

**Results:**

The highest fungal load was in the cold season, with *Aspergillus* sp. being the predominant species, and the highest bacterial load in the warm season. Reduced susceptibility to azoles was observed in both seasons, with greater species diversity in the cold season. In the warm season, *Nidulantes* and *Fumigati* sections of *Aspergillus* were detected by RT-PCR, with *Fumigati* being the most prevalent; in the cold season, only *Nidulantes* was detected. Mycotoxins, mainly fumonisins, were more prevalent in the warm season; in the cold season, griseofulvin was the most prevalent mycotoxin. Cytotoxicity was more prevalent in A549 cells than in SK cells. Settled dust caused greater cytotoxicity in SK cells, and filters from the vacuumed dust in A549 cells. Higher particulate matter concentrations in the indoor sampled areas suggest a significant contribution of indoor activities to workers’ exposure.

**Discussion:**

The study highlights concern about seasonal variations in microbial contamination, emphasizing the potential for respiratory diseases, invasive infections by azole-resistant fungi, mycotoxin exposure, and cytotoxicity in lung cells due to co-exposure to fungi, particulate matter, and mycotoxins influenced by environmental conditions.

## 1 Introduction

Workers in the wood industry may be exposed to several occupational hazards that can cause adverse health effects, including microbial agents such as fungi and bacteria (1, 2). *Aspergillus*, *Penicillium,* and *Cladosporium* are the most common fungal genera found in this industry (1). The impact of seasonal variations on the microbial composition of indoor air, including these fungal genera, has been demonstrated (1, 3). Therefore, to assess indoor microbial concentrations it is important to consider the influence of season-related parameters, such as indoor and outdoor air temperature and relative humidity (3).

Most *Aspergillus* species are found in a variety of indoor habitats, with some of them being the cause of several opportunistic human infections (4–6). *Aspergillus* conidia small and specific size range allow them to be easily diffused in the air (being found anywhere) and to be inhaled by and colonize the upper and lower respiratory tract of those exposed (6, 7). In carpentries, occupational exposure by inhalation to airborne fungi may occur due to the high prevalence of wood dust, which is classified as carcinogenic (Group 1) to humans by the International Agency for Research on Cancer (IARC) (8–10), since wood dust acts as a carrier of several agents, including microorganisms and their metabolites (e.g., mycotoxins), to the respiratory system of workers (11).

Recently, the WHO developed a priority list of fungal species with pathogenic potential, in response to the rising threat of fungal infections and antifungal resistance. The WHO has identified three priority groups of fungal species, of critical, high, and medium risk, based on numerical scores and expert consensus (12). *Aspergillus fumigatus*, which integrates the WHO critical group, was recently identified and widespread in another woodworking environment in Portugal, in “Do It Yourself” (DIY) stores (13). *Aspergillus* sp. and other fungal species, such as *Penicillium*, *Alternaria*, and *Fusarium*, can produce mycotoxins (1, 14–16). Mycotoxins can endure harsh environmental conditions (such as high or low temperatures) and therefore remain in the environment for a long time even in the absence of producing fungi (1, 14, 17). The primary concern regarding mycotoxins lies in their ubiquitous presence in various environments and their significant toxicological impact on human health (16), and to the authors’ knowledge, there are no studies reporting the assessment of mycotoxins in carpentries (1).

The wood industry in Portugal comprises approximately 16,600 companies that employ around 80,000 people (data from 2021) (18). Depending on the size of the carpentry, the timber can be stored outside, or inside which can be influenced by exposure to environmental factors such as temperature, humidity, and air circulation. When stored outdoors, wood is exposed to higher fluctuations in these environmental conditions, especially increased humidity and moisture; when stored indoors, storage provides more controlled conditions, generally leading to reduced microbial growth due to less humidity and more stable environmental conditions. However, indoor environments can still enhance microbial contamination if not properly ventilated or cleaned, posing a threat to the health of workers (19).

On the other hand, indoor storage provides more controlled conditions, generally leading to reduced microbial growth due to less moisture and more stable environmental factors. However, indoor environments can still harbor microbial contamination if not properly ventilated or cleaned.

Most of these companies are small and family-owned, and frequently face challenges in preventing occupational injuries and illness because they lack resources, do not employ personnel specifically focused on safety and health, and are often unable to detect and monitor hazards in the workplace (20).

Therefore, the main goal of this study is to analyse the microbial contamination in Portuguese carpentries through complementary sampling and analytical methods, in different seasons, in order to support actions that promote safe and healthy working conditions in this sector. To this end, bacterial and fungal contamination, fungal susceptibility to azoles, mycotoxins, particulate matter (PM), and the *in vitro* cytotoxicity of the mixture of contaminants collected by passive sampling were measured, and then related with the used sampling method, sampling location and seasonal variations.

## 2 Materials and methods

### 2.1 Selection and characterization of the carpentries

**Figure 1:**
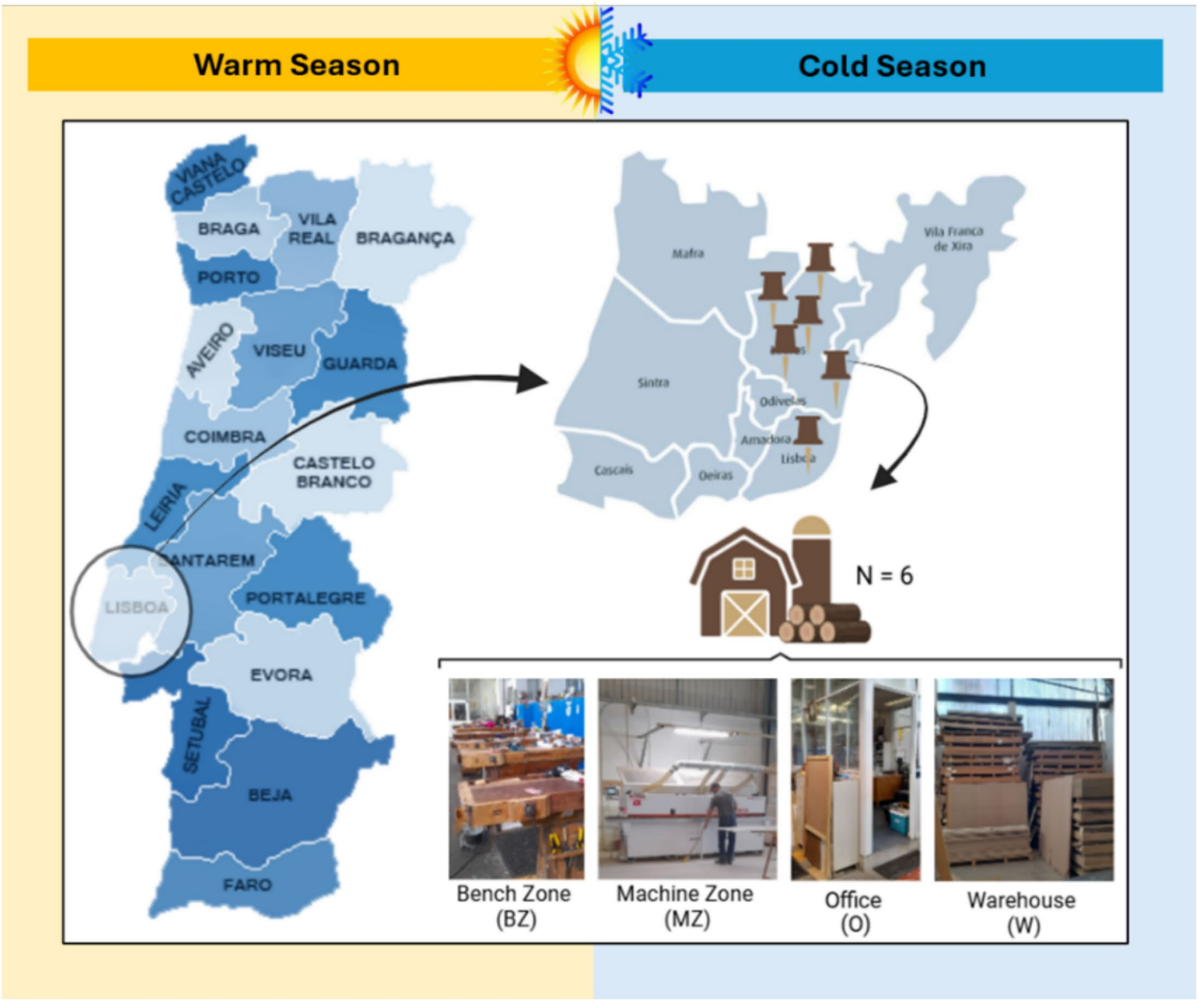
Geographical distribution of evaluated carpentries and sampling sites.

During the warm and cold seasons - between June–July and October–November 2023, respectively, - a total of 6 carpentries located in the Lisbon Metropolitan Area, Portugal, (specifically in the cities of Loures and Lisboa), were selected for a sampling campaign conducted by researchers/exposure assessors over an eight-hour work shift. Microbial occupational exposure was assessed in the bench zone (BZ), machine zone (MZ), warehouse (W), and office (O) (Figure 1).

When there was no physical division between BZ and MZ, sampling was done in a single place identified as BZ/MZ. These sampling sites were chosen considering the carpentry layout. The bench zone (BZ) is the area where workers, for example, assemble furniture or varnish the wood. The machine zone (MZ) is where machines are located, such as the wood-cutting machines. The warehouse (W) is the storage location for materials such as unprocessed wood or wood application products. The office (O) is the administrative location of the carpentry. As a reference, outdoor air samples (E) were also collected.

The main tasks carried out by carpentry workers are based on the assembly, repair, or restoration of furniture, which includes preparing the product, measuring and working the wooden pieces until the final product. The cleaning process for each area was usually carried out by workers once a week, in places with the most accumulated dust, where workers normally used brooms and air compressors to clean.

In all carpentries, a walkthrough survey (Supplementary Table S1) was carried out to collect data relating to the number of workers, characteristics of the ventilation system, type and source of wood, use of personal protective equipment (PPE), cleaning procedures, management of the wood waste, and temperature of workplaces.

### 2.2 Sampling campaign

#### 2.2.1 Active sampling

Two impaction air samplers, the MAS-100 air sampler (Millipore, Billerica, MA, United States), and the Andersen six-stage cascade impactor (Thermo Fisher Scientific, Waltham, MA, United States), were used to measure the microbial load in both seasons. An impingement air sampling method, the Coriolis *μ* air sampler (Bertin Technologies, Montigny-le Bretonneux, France), was also used during the warm season campaign (due to a technical issue, its use during the cold season campaign was not possible) to analyze the presence of mycotoxins.

**Figure 2:**
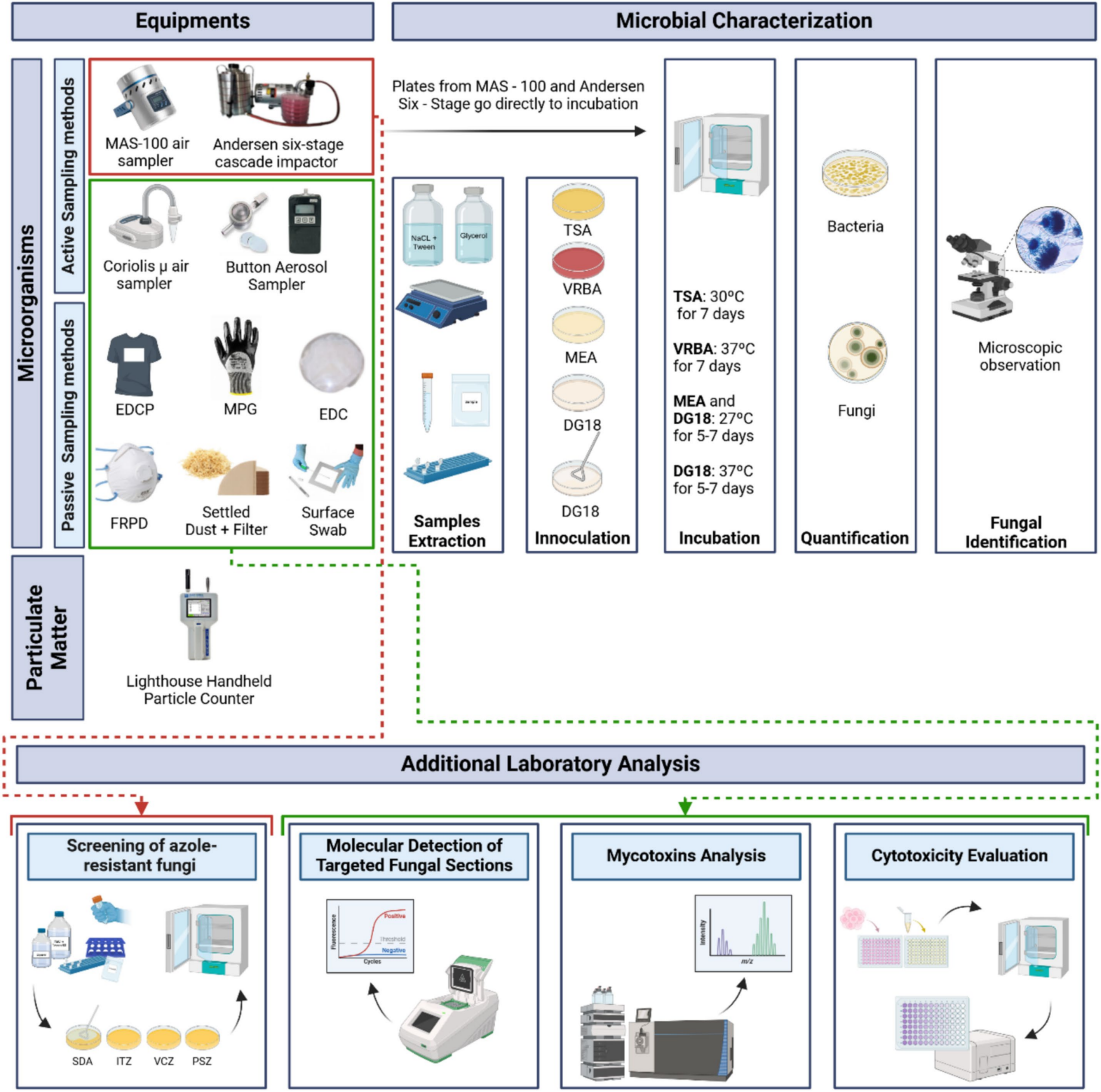
Sampling strategy applied and analysis flowchart of the environment samples collected (Created in BioRender. Dias, M. (2025) https://BioRender.com/zb6iedz).

Active sampling methods were used at all sampling sites. Additionally, an outdoor sample (E) was collected with a MAS-100 air sampler and a Coriolis *μ* air sampler to be used as a reference. For personal air sampling, an SKC Button Aerosol Sampler with a 0.8 μm 25 mm polycarbonate filter, connected to an SKC air sampling pump was applied in only two workers from each carpentry (one from BZ and one from MZ) for 2 h, except for the carpentries, which did not have a separate machine area and bench area, in which case only one worker used the personal air sampler (Figure 2). Sampling details are described in Supplementary Table S2.

#### 2.2.2 Passive sampling

Several passive sampling methods were used, namely, Electrostatic Dust Collectors (EDC), settled dust (SD) obtained via vacuuming, filters from vacuumed dust, floor surface swabs, mechanical protection gloves (MPG) and filtering respiratory protection devices (FRPD) worn by workers, and Electrostatic Dust Collectors—Personal (EDCP), attached to workers’ t-shirt aiming to assess the accumulation of microorganisms that goes to their clothes (21) (Figure 2). Although filtering respiratory protection devices (FRPDs) were included in the sampling strategy, they were not being used by the workers at the time of sampling; therefore, none were available for collection or analysis. Passive sampling was carried out by all described methods in all sampling sites, except the EDCP which was placed on two workers, one from the bench zone and the other from the machine zone during a work shift (8 h). Before analysis, all samples were kept refrigerated (0–4°C) in sterile bags. Sampling procedures are presented in Supplementary Table S2. Passive samples were extracted as previously described (22) (Supplementary Table S3).

### 2.3 Characterization of viable microbiota

The culture media used for active and passive samples were: malt extract agar (MEA) supplemented with chloramphenicol (0.05%) incubated at 27°C, and dichloran-glycerol agar (DG18) incubated at 27°C and 37°C to evaluate the pathogenic potential of fungi, for fungi; and tryptic soy agar (TSA) supplemented with nystatin (0.2%) incubated at 30°C, and violet red bile agar (VRBA) incubated at 37°C, for bacteria. In the case of the MAS-100 air sampler, the culture media used for fungi were MEA and DG18 (both incubated at 27°C) whereas on Andersen six-stage cascade impactor, due to its ability to sample by particle size, it was used DG18 twice to allow the incubation at two temperatures as mentioned above.

The screening of azole-resistant fungi was carried out as previously described (22) adapted from EUCAST guidelines (23, 24), with azole-supplemented Sabouraud agar plates incubated for 48 h at 27°C. In order to quantify colony-forming units (CFU) and CFU concentration (CFU.m^−3^/m^−2^/m^−2^.day^−1^/g^− 1^) appropriate formulas were used as described in previous studies (25). For the fungal morphological identification, macro and microscopic characteristics were identified by an environmental and occupational mycologist, and the fungi were identified to the genus level.

### 2.4 Molecular detection of targeted fungal sections

In all passive samples except for surface swabs, molecular detection of *Aspergillus* sections *Fumigati, Nidulantes* and *Circumdati* was carried out by Real-Time PCR (qPCR). Fungal DNA was extracted from the samples with ZR Fungal/Bacterial DNA MiniPrep Kit (Zymo Research, Irvine, USA) and analyzed in CFX-Connect PCR System (Bio-Rad). The sequence of primers and TaqMan probes as well as the amplification details (22) are described in Supplementary Tables S4, S5, respectively. As controls, a non-template control was used as a negative control, and DNA from a reference kindly conceded by the Reference Unit for Parasitic and Fungal Infections, Department of Infectious Diseases, National Health Institute Doctor Ricardo Jorge, IP, as positive control. These strains have been sequenced for ITS, B-tubulin, and Calmodulin.

### 2.5 Mycotoxin analysis

Thirty-eight mycotoxins were investigated in a total of 132 samples, including 19 Coriolis a *μ* air samples, 36 SD, 30 filters used in the vacuum cleaner, 25 EDC, 21 EDCP, and 1 MPG recovered during the warm season. Mycotoxin detection was performed using a Nexera high-performance liquid chromatograph (HPLC) (Shimadzu, Tokyo, Japan) equipped with a mass spectrometry detector (5,500 Qtrap) (Sciex, Foster City, CA, United States). The analysis was developed according to a pre-established protocol (22). Procedure details and Limits of Detection (LOD) of each mycotoxin are provided in the Supplementary Tables S6, S7.

### 2.6 Cytotoxicity evaluation

To measure the cytotoxicity of contaminants present in carpentries from Coriolis air, settled dust, vacuum cleaner filters, EDC, EDCP, and MPG, two-fold serial dilutions of samples were prepared and incubated with human lung (A549) and swine kidney (SK) epithelial cells. The impact of samples’ contaminants in the metabolic activity of cells was assessed using the 3-(4,5-dimethylthiazol-2-yl)-2,5-diphenyltetrazolium bromide (MTT) as previously reported (22), with the details described in Supplementary methods S1.

### 2.7 Particulate matter

To monitor the particulate matter, temperature, and humidity, the Lighthouse Handheld Particle Counter HH3016-IAQ was used. This monitoring was conducted in 4 different sites – BZ, MZ, O and W. The measurements details are described in Supplementary Table S8.

Supplementary Table S9 summarizes the number of samples/measures from each sampling method used in all carpentries, as well the recovered MPG.

### 2.8 Statistical analysis

The data were analyzed using the statistical software R-Studio, version 4.3.3. The results were considered significant at a 5% significance level. Descriptive and Exploratory Data Analysis was used to characterize the sample. To test the normality of the data, the Shapiro–Wilk test was used. Regarding particulate matter, fungi and bacteria in Andersen six-stage cascade impactor, Principal Component Analysis (PCA) was carried out to reduce information. PCA was applied on the correlation matrix after the standardization of data. The components were extracted forcing independence using the Varimax rotation with Kaiser normalization to ensure orthogonality. To evaluate the quality of the PCA, the Kaiser-Meyer-Olkin statistic (KMO = 0.636, revealing a reasonable quality) and the Bartlett test for sphericity were used [having rejected the hypothesis that the correlation matrix was the identity matrix (*p* < 0.05)]. To study the relationship between fungal contamination, bacterial contamination, resistance to azoles, particulate matter and environmental conditions, Spearman’s correlation coefficient was used, since the assumption of normality was not verified. To compare fungal contamination, bacterial contamination, resistance to azoles and particulate matter between summer and winter, the Mann–Whitney test was used, since the assumption of normality was not met.

## 3 Results

### 3.1 Microbial contamination

#### 3.1.1 Microbial distribution by sampling site

Regarding active sampling, MAS-100 presented the highest fungal and bacterial load in both seasons (Supplementary Table S10).

The quantitative comparison (indoor/outdoor) of the fungal load in the warm season showed BZ and MZ with higher indoor fungal load on MEA, and BZ with higher indoor fungal load on DG18. In the cold season, all sampling sites showed lower indoor fungal load on MEA, whereas BZ, MZ, and W showed higher indoor fungal load on DG18. Regarding bacteria, in the warm season all sampling sites showed a higher indoor bacterial load on TSA, and BZ showed a higher indoor bacterial load on VRBA. In the cold season, BZ showed a higher indoor bacterial load on TSA, whereas BZ, MZ and W showed a higher indoor bacterial load on VRBA (Supplementary Table S10). Overall, considering passive sampling methods, surface swabs presented the highest fungal and bacterial contamination in both seasons, followed by filters used on the vacuum cleaner (Supplementary Table S11).

#### 3.1.2 Fungal distribution by sampling method

**Figure 3:**
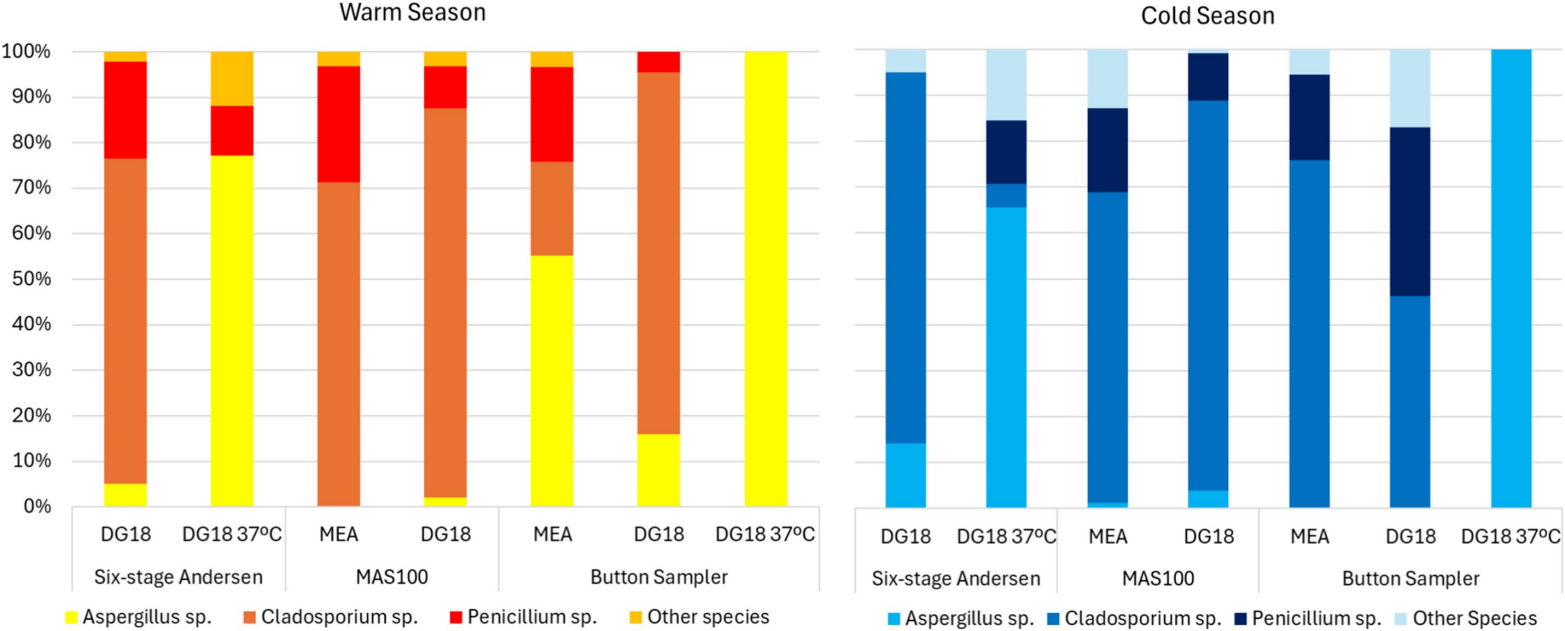
Species diversity on active sampling methods.

Active sampling in the warm season found the highest prevalence of *Aspergillu*s sp. in the following conditions: on DG18 37°C, Button Sampler (100.0%) and six-stage Andersen (77.1%); on DG18 27°C, Button Sampler (15.9%) and six-stage Andersen (5.0%); on MEA, Button Sampler (55.2%). The same trend was observed in the cold season, with the highest prevalence of *Aspergillu*s sp. on DG18 37°C, Button Sampler (100.0%) and six-stage Andersen (65.5%) (Figure 3).

**Figure 4:**
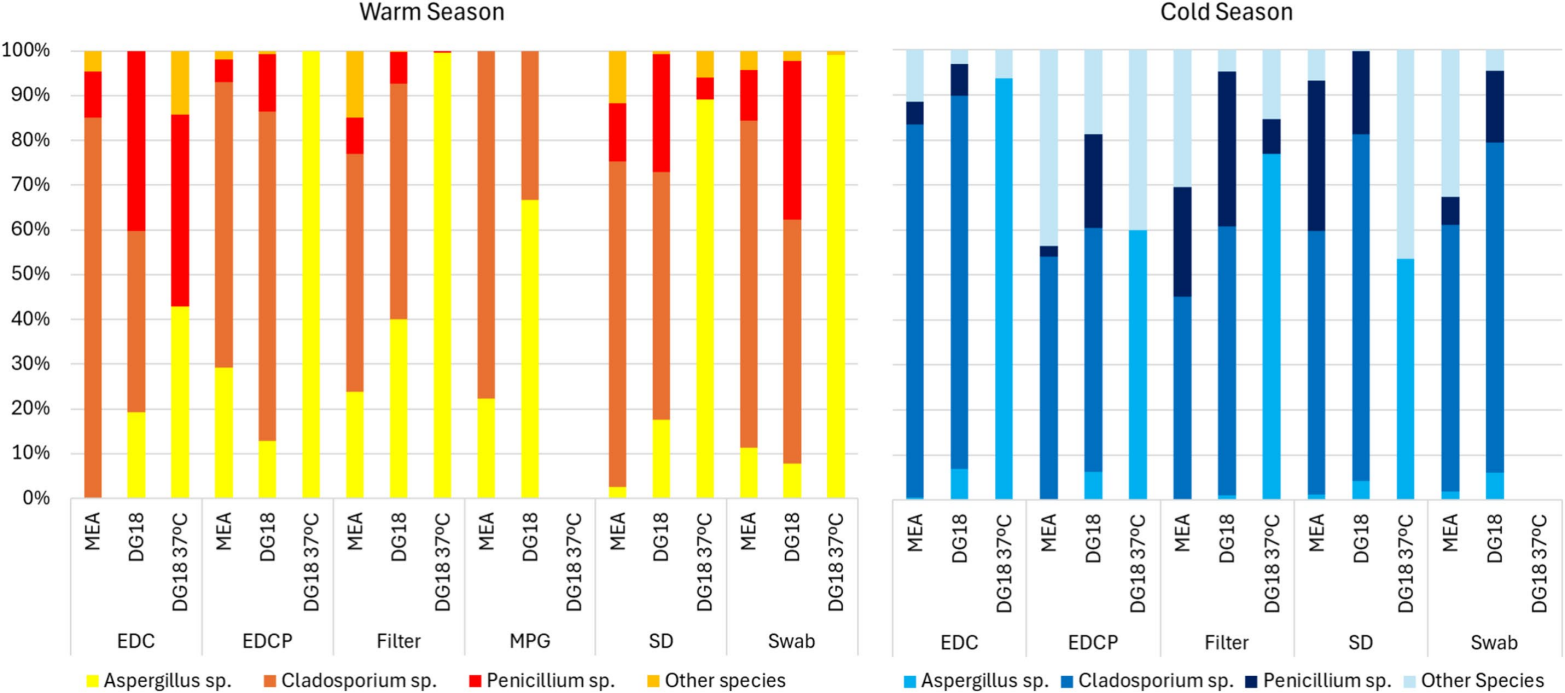
Genera distribution on passive sampling methods.

Regarding passive sampling, in the warm season, *Aspergillus* sp. presented the highest prevalence on DG18 incubated at 37°C (EDC: 42.9% | EDCP: 100.0% | Filters: 99.6% | SD: 89.1% | Swabs: 99%). In the cold season, *Aspergillus* sp. also presented the highest prevalence on DG18 incubated at 37°C (EDC: 93.8% | EDCP: 60.0% | Filters: 76.9% | SD: 53.6%) (Figure 4).

#### 3.1.3 Screening of antifungal resistance

In the warm season, reduced susceptibility to tested azole concentrations was observed for *Mucor* sp. (ITZ: 60.0%; VCZ: 66.7%; PSZ: 100.0%), *Cladosporium* sp. (ITZ: 40.0%), and *Chrysonilia* sp. (VCZ: 33.3%). In the cold season, reduced susceptibility was observed for *Penicillium* sp. (ITZ: 21.1%; VCZ: 30.0%; PSZ: 35.7%), *Cladosporium* sp. (ITZ: 42.1%; VCZ: 40.0%; PSZ: 35.7%), *Mucor* sp. (ITZ: 15.8%; VCZ: 20.0%; PSZ: 14.3%), *Alternaria* sp. (VCZ: 6.7%; PSZ: 7.1%), *Chrysonilia* sp. (ITZ: 5.3%; VCZ: 3.3%; PSZ: 7.1%) and *Chrysosporium* sp. (ITZ: 10.5%) (Table 1). In non-supplemented SDA media (used as control), *Cladosporium* sp. was the most frequent in the warm season (82.4%) and in the cold season in *ex aequo* with *Penicillium* sp. (31.8% each). One isolate of *Aspergillus* section *Nigri* was observed in non-supplemented SDA in the cold season (Table 1).

**Table 1:** Fungal distribution per azole- supplemented media.

Season	Fungi	SDA* (CFU)	%	ITZ* (CFU)	%	VCZ* (CFU)	%	PSZ* (CFU)	%
Warm season	*Cladosporium* sp.	42	82.4	2	40.0				
	*Mucor* sp.	6	11.8	3	60.0	6	66.7	4	100
	*Penicillium* sp.	2	3.9						
	*Rhizopus* sp.	1	1.9						
	*Chrysonilia* sp.					3	33.3		
	Total	51	100	5	100	9	100	4	100
Cold season	*Penicillium* sp.	14	31.8	4	21.1	9	30,0	5	35.7
	*Cladosporium* sp.	14	31.8	8	42.1	12	40,0	5	35.7
	*Chrysosporium* sp.	4	9.0	2	10.5				
	*Alternaria* sp.	3	6.8			2	6,7	1	7.1
	*Mucor* sp.	3	6.8	3	15.8	6	20,0	2	14.3
	*Chrysonilia* sp.	2	4.6	1	5.3	1	3,3	1	7.1
	*Paecilomyces* sp.	1	2.3						
	*Aspergillus* sp.	1	2.3						
	*Fusarium* sp.	1	2.3	1	5.2				
	*Trichoderma* sp.	1	2.3						
	TOTAL	44	100	19	100	30	100	14	100

* SDA, sabouraud dextrose agar; ITZ, itraconazole; VCZ, voriconazole; PSZ, posaconazole.

The isolates with reduced susceptibility to azoles were recovered from EDC, SD and filters from the vacuum cleaner in both seasons, and additionally from EDCP in the warm season.

### 3.2 Molecular detection targeting *Aspergillus* fungal sections

The molecular detection results of three *Aspergillus* sections were different in each season for all passive sampling methods, except for surface swabs. In the warm season, two *Aspergillus* sections were detected: *Aspergillus* section *Nidulantes* in 2 SD samples (12.5%), and in 2 filters from the vacuum cleaner (12.5%), *Aspergillus* section *Fumigati* in 3 BS (27.3%), 9 filters from the vacuum cleaner (56.3%), and 13 settled dust samples (81.3%). Overall, *Aspergillus* section *Nidulantes* was detected but not identified through culture-based methods in 4 out of 4 samples (100.0%). *Aspergillus* section *Fumigati* was detected but not identified through culture-based methods in 21 out of 25 samples (84.0%) (Supplementary Table S12).

In the cold season, only *Aspergillus* section *Nidulantes* was detected, in 2 SD samples (15.4%) and in 2 filters from the vacuum cleaner (12.5%). *Aspergillus* section *Nidulantes* was detected but not identified through culture-based methods in 4 out of 6 samples (66.7%) (Supplementary Table S12).

### 3.3 Mycotoxin contamination

**Figure 5:**
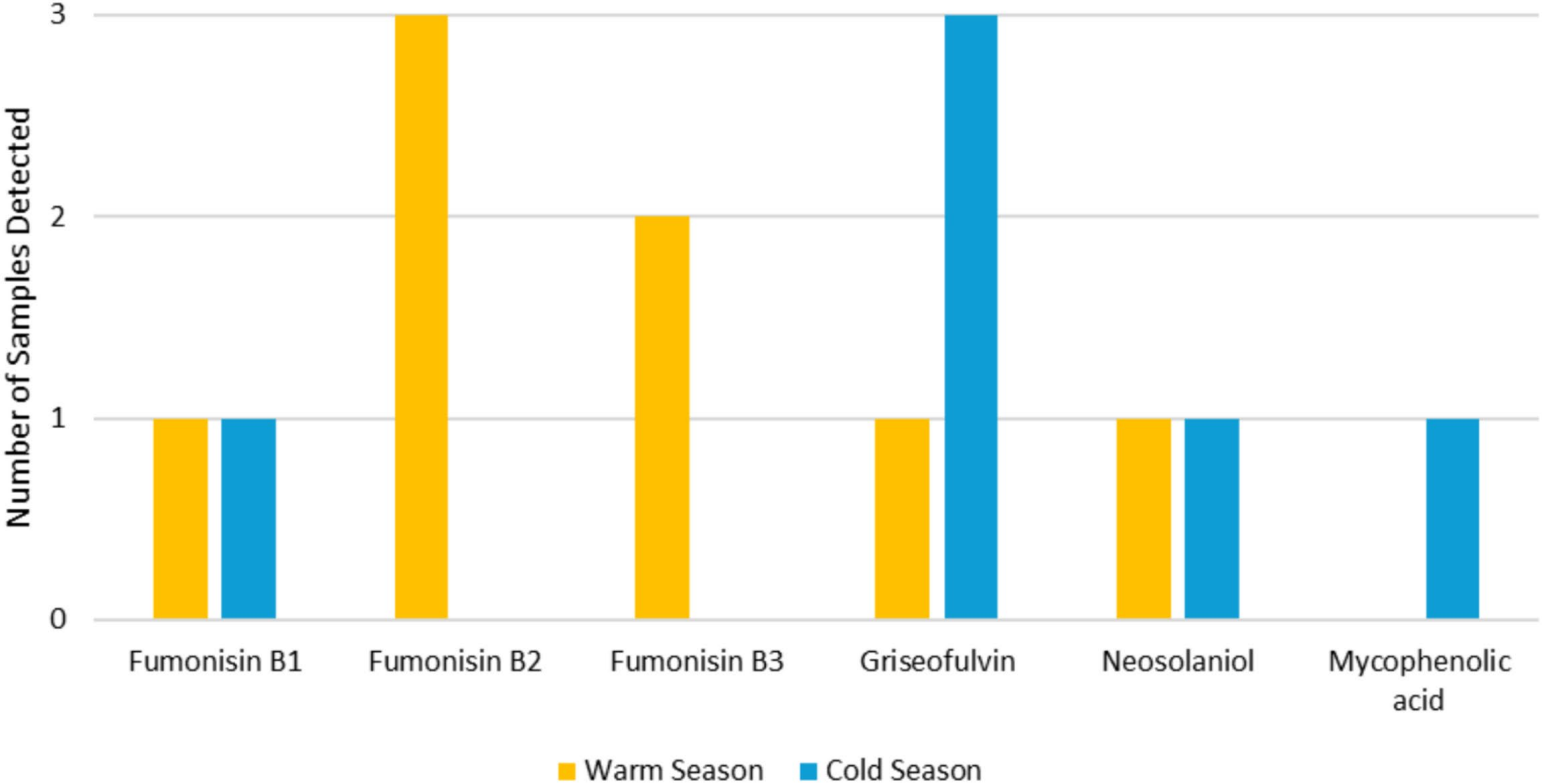
Mycotoxin’s prevalence in both seasons.

In the warm season, mycotoxins were detected in 4 out of 20 settled dust samples (20.0%) and in 1 out of 11 EDCP (9.1%). Fumonisin B2 and B3 were the most detected mycotoxins found in 2 out of 20 samples (10.0%) with values <LOQ (25.0 ng/g) and <LOQ (27.9) respectively. This was followed by fumonisin B1, griseofulvin and neosolaniol in 1 out of 20 samples (5.0%) (different samples) with values <LOQ (40.8 ng/g), <LOQ (8.7) and 12.1 ng/g, respectively. In one of the settled dust samples, the three fumonisin (B1, B2 and B3) were found, and in another one, both fumonisin B2 and B3 were found. On the EDCP, the only mycotoxin found was fumonisin B2, with a value of <LOQ (25.0 ng/g). Considering the cold season, mycotoxins were also detected in 4 settled dust samples (25.0%) and in 1 EDCP (10.0%). Griseofulvin was the most detected mycotoxin, found in 3 out of 16 samples (18.8%) with values ranging from <LOQ (8.7 ng/g) to 1,090 ng/g. This was followed by mycophenolic acid and neosolaniol in 1 out of 20 samples (5.0%) (different samples) with values of 132 ng/g and 16.9 ng/g, respectively. In one of the settled dust samples, both griseofulvin and mycophenolic acid were found (Figure 5).

### 3.4 Cytotoxicity evaluation

Five serial 1:2 dilutions of samples’ extracts were tested towards SK and A549 cells. Cellular viability was measured at 510 nm and three cytotoxicity levels (low, moderate, high) were defined based on sample dilution (Table 2). In the warm season, some level of cytotoxicity was observed with 50.6% of samples in lung epithelial A549 cells, and with 37.7% of samples in SK renal cells. Moderate to high toxicity was observed for settled dust and gloves (*N* = 17) in SK cells [samples recovered from the BZ (*n* = 7), MZ (*n* = 6), W (*n* = 2), and BZ/MZ (*n* = 1)], and for settled dust, gloves and EDCP (*N* = 18) in A549 cells [samples recovered from the BZ (*n* = 12), MZ (*n* = 10), W (*n* = 2), and BZ/MZ (*n* = 2)]. In the cold season, some level of cytotoxicity was observed with 84.9% of samples in A549 cells, and with 34.0% of samples in SK cells. In SK cells, settled dust accounted for 66.6% of the observed cytotoxicity [samples recovered from the BZ (*n* = 4), MZ (*n* = 6), and W (*n* = 2)]. In A549 cells, vacuum filters accounted for 31.1% of the observed cytotoxicity [samples recovered from the BZ (*n* = 5), MZ (*n* = 5), W (2) and O (*n* = 2)], and settled dust accounted for 28.9% of the observed cytotoxicity.

**Table 2:** Toxicity level of filters, EDC, EDCP, masks, gloves, Coriolis and settled dust in SK and A549 cells.

	SK				A549			
	High	Moderate	Low	(−)	High	Moderate	Low	(−)
Warm season								
Filters (*N* = 16)	0	0	4	12	0	0	8	8
EDC (*N* = 16)	0	0	5	11	0	0	9	7
EDCP (*N* = 9)	0	0	3	6	0	1	4	4
Gloves (*N* = 1)*	0	1	0	0	0	1	0	0
Coriolis (*N* = 19)*	0	0	0	19	0	0	0	19
Settled dust (*N* = 16)	3	13	0	0	6	10	0	0
	3 (3.9%)	14 (18.1%)	12 (15.6%)	48 (62.3%)	6 (7.8%)	12 (15.6%)	21 (27.3%)	38 (49.4%)
Cold season								
Filters (*N* = 16)	0	0	1	15	4	3	7	2
EDC (*N* = 16)	0	0	5	11	0	0	11	5
EDCP (*N* = 8)	0	0	0	8	0	2	5	1
Settled dust (*N* = 13)	0	7	5	1	1	7	5	0
	0 (0%)	7 (13.2%)	11 (20.8%)	35 (66.0%)	5 (9.4%)	12 (22.6%)	28 (52.8%)	8 (15.1%)

Semi-quantitative scale for cytotoxicity grading was adopted: low cytotoxicity (+) with IC50 values ranging from 31.251 cm2/ml to 7.813 cm2/ml; medium cytotoxicity (++) with IC50 values ranging from 3.906 cm2/ml to 0.977 cm2/ml; high cytotoxicity (+++) with IC50 values ranging from 0.488 cm2/ml to 0.061 cm2/ml. The absence of cytotoxicity was considered when the extract concentration at 31.251 cm2/ml failed to inhibit the growth of the swine kidney and A549 cells. A549, human alveolar epithelial cells; SK, swine kidney cells; EDC, Electrostatic dust collector; High, IC50 ≥ 3rd dilution; Moderate, IC50 at 2nd dilution; Low, IC50 at 1st dilution; (−), no cytotoxicity. * Only sampled in the warm season.

### 3.5 Particulate matter

In the warm season, particulate matter levels were higher in the bench zone (BZ) for some particle diameters (1.0 *μ* – 151.6 μg/m^3^; 5.0 μm – 3849.6 μg/m^3^; 10.0 μm – 5861.1 μg/m^3^). Regarding the I/O ratio, for PM2.5, PM5, and PM10, BZ, MZ and W had an I/O ratio >1 indicating that increased concentrations indoors in these sampling sites are caused by indoor emissions sources (e.g., activities developed). In the cold season, particulate matter levels were higher in the machine zone (MZ) for most particle diameters (0.5 μm – 55.0 μg/m^3^; 1.0 μ – 655.3 μg/m^3^; 2.5 μm – 3636.5 μg/m^3^; 5.0 μm – 9690.9 μg/m^3^; 10.0 μm – 16185.0 μg/m^3^). Regarding the I/O ratio, for PM2.5, PM5, and PM10, also BZ, MZ, and W had an I/O ratio >1 indicating that increased concentrations indoors in these sampling sites are also caused by emissions sources indoors.

**Figure 6:**
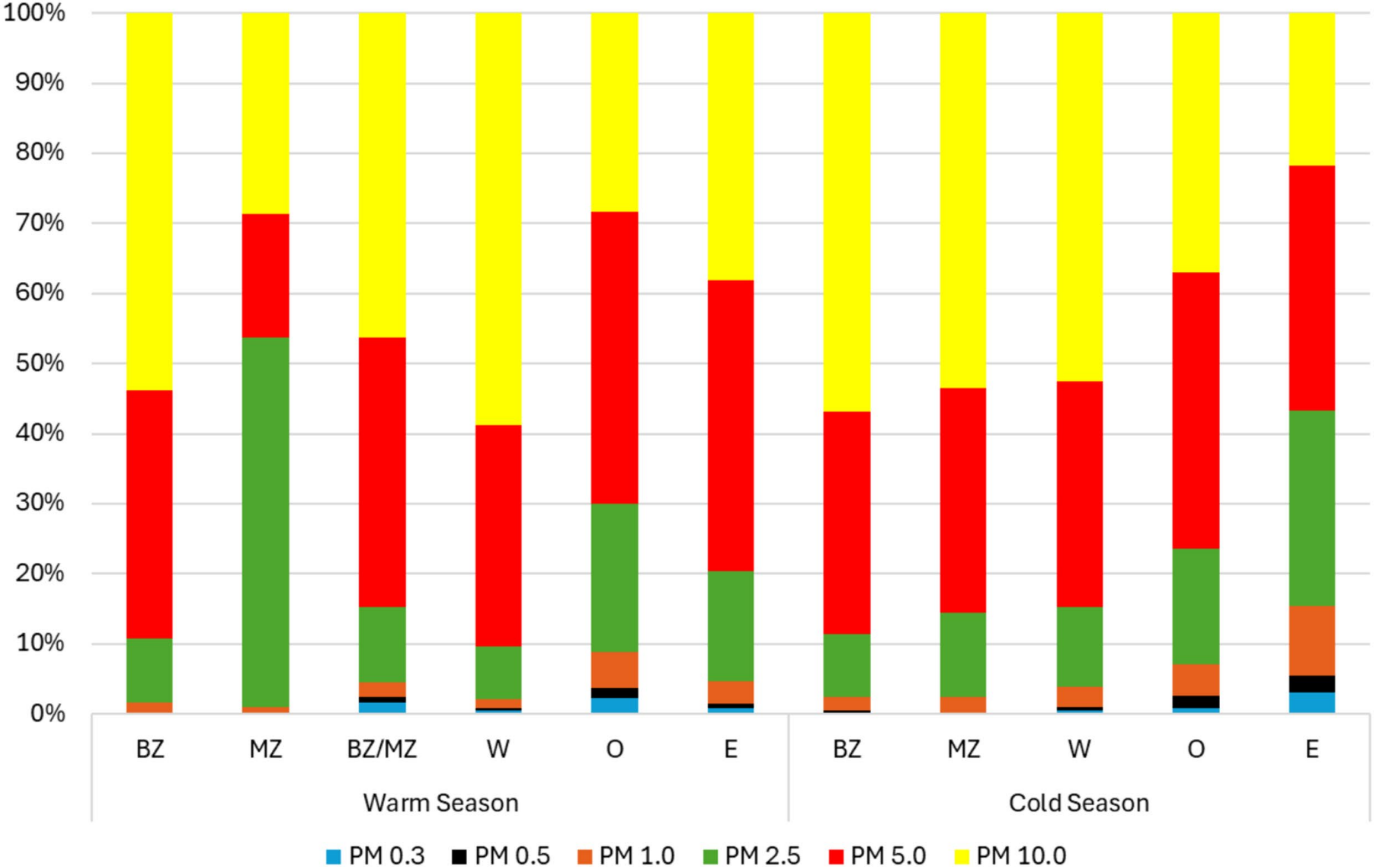
Particulate matter prevalence in each site during both seasons.

Regarding the distribution of each particle diameter, in the warm season, PM10 presented the highest prevalence in most sampling sites (BZ – 53.9%; BZ/MZ – 46.4%; W – 58.9%) followed by PM5 (BZ-35.4%; BZ/MZ – 38.3%; W – 31.5%). The outdoors presented the highest prevalence of inhalable particle sizes PM5 (41.4%) followed by PM10 (38.2%). Considering the cold season, the same distribution was observed (Figure 6).

### 3.6 Correlation analysis

#### 3.6.1 Principal component analysis

##### 3.6.1.1 Particulate matter and environmental conditions

In the warm season, particle counts of various sizes showed strong positive correlations with each other, with the exception of particles measuring 0.3 *μ* and 2.5 μ. No significant correlations were observed between particle counts and environmental factors such as temperature or relative humidity (as shown in Supplementary Figure S1). In the cold season, particle counts across different sizes were also strongly correlated, except for particles of size 0.3 μ. The only significant environmental relationship identified was for 0.3 μ particles, where higher counts were associated with higher relative humidity (r_S_ = 0.673, *p* = 0.001) (Supplementary Figure S1). From the PCA, two components were extracted that explain 75.9% of the global variance, component 1 consisting of particles 0.5, 1.0, 5.0, and 10.0 with 57.4% of the variance and component 2, consisting of particles 0.3 and 2.5 with 18.5% of variance. These two components were used in the correlation and comparison analysis.

##### 3.6.1.2 Andersen six-stage cascade impactor

Regarding to Andersen six-stage cascade impactor and considering the strong correlations obtained (Supplementary Table S13) between fungal and between bacterial contamination in the different stages, PCA was carried out, to reduce the number of variables, and the following results were obtained:

- For fungal contamination in DG18, two components that retain 79.5% of the total variance, component 1 consisting of stages 2 to 5 (with 44.2% of the global variance) and component 2 consisting of stages 1 and 6 (with 35.3% of the global variance);
- For fungal contamination in DG18 37°C, also two components that retain 67.2% of the total variance, component 1 consisting of stages 1 to 4 (with 44.2% of the global variance) and component 2 consisting of stages 5 and 6 (with 23.0% of global variance);
- For bacterial contamination in TSA, also two components that retain 79.7% of the global variance, component 1 consisting of stages 1 to 4 (with 40.9% of the global variance) and component 2 consisting of stages 5 and 6 (with 38.8% of global Variance);
- For bacterial contamination in VRBA, only one component was obtained, meaning that the 6 stages were considered as a linear combination resulting from PCA.

#### 3.6.2 Comparison between warm and cold seasons

No statistically significant differences were detected in particle concentration (p’s > 0.05) between the warm and cold seasons (Supplementary Table S14); however, some correlations were observed regarding microbial contamination in different sampling methods (Table 3).

**Table 3:** Results by season (microbial contamination and PM).

		Warm season	Cold season
Microbial contamination	Active Sampling Methods		Higher fungal load (DG18) on Andersen six-stage cascade impactor (stage 6) Higher fungal (DG18) and bacterial (TSA) load on MAS-100 Higher fungal (DG18) and bacterial (TSA) load on Button Air Sampler
	Passive Sampling Methods	Higher fungal contamination (DG18 incubated at 37°C) in surface swab samples Higher fungal contamination (SDA) in EDCP samples Higher fungal contamination (SDA) in filters from the vacuum cleaner	Higher fungal contamination (MEA) in EDC samples Higher fungal contamination (DG18) and higher fungal resistance (ITZ, VCZ and PSZ) in surface swab samples Higher fungal resistance (VCZ) in SD samples Higher bacterial contamination (TSA) in filters from the vacuum cleaner samples
Particulate matter		MZ presented a higher concentration of particles from size 0.5 μ than E BZ and MZ presented a higher concentration of particles from size 2.5 *μ* and 10.0 μ than E BZ presented a higher concentration of particles from size 5.0 μ than E	MZ presented a higher concentration of particles from size 1.0 μ, 2.5 μ, 5.0 μ and 10.0 μ than E

EDC, Electrostatic Dust Cloth; EDCP, e-cloth; SD, Settled Dust; MZ, Machine Zone; BZ, Bench Zone; E, Exterior; MEA, Malt Extract Agar; DG18, Dicloran-Glycerol Agar; TSA, Tryptic Soy Agar; SDA, Saboraud Dextrose Agar; ITZ, Itraconazole; VCZ, Voriconazole; PSZ, Posaconazole.

Regarding the comparison of the concentration of particulate matter between monitoring sites, only the BZ, MZ and E sites were considered, as the remaining sites did not have a sufficient number of observations for this analysis. No statistically significant differences were detected between BZ and MZ in both seasons however, some differences were detected between indoor (BZ and MZ) and outdoor (E) concentrations of particles (Table 1 and Supplementary Figures S2–S14).

## 4 Discussion

Microbial contamination poses a significant threat to public health, particularly in occupational settings where workers may be exposed for long periods and continuously to several microorganisms with pathogenic potential (26). While microbial contamination naturally varies with seasonality, climate change can significantly alter the prevalence and persistence of critical pathogens (27). In woodworking environments, there has been a long association with an increased risk of respiratory symptoms and diseases, including asthma, chronic bronchitis, hypersensitivity pneumonitis, and other allergic or irritative conditions (28–30), largely due to prolonged exposure to wood dust and microbial contaminants such as fungi and bacteria (13).

To contextualize the present findings, it is important to compare them with previous studies conducted in similar occupational environments. Several investigations have explored microbial contamination and organic dust exposure among woodworkers across different geographical regions (31–36). Alwis et al. (31) and Mandryk et al. (32) reported high levels of bacterial and fungal contamination in sawmills, supporting the idea that microbial exposure is a widespread issue in woodworking industries. Similarly, Spee et al. (36) and Schlünssen et al. (33) found associations between wood dust exposure and respiratory symptoms, consistent with the potential health risks identified in the present study. The presence of pathogenic fungal species and organic dust reported by Mackiewicz et al. (34) and Wójcik-Fatla et al. (35) further corroborates the relevance of this topic and the need for ongoing monitoring in this occupational sector. Compared to these studies, our research contributes novel insights by incorporating seasonal variation, assessing mycotoxins presence, and evaluating cytotoxicity in both lung and liver cell lines. These results, which will be further discussed provide a more comprehensive understanding of the occupational hazards faced by woodworkers.

This study focused on the assessment of microbial contamination in two seasons (warm and cold season), using complementary sampling methodology that applies both active and passive sampling methods, to overcome the disadvantages of each method, for a complete and adequate characterization of the microbial risks to which these workers are exposed (1). Active sampling methods rely mostly on culture-based methods which can be beneficial or not depending on the goal of each study. For instance, microorganisms’ inflammatory or cytotoxic potential may be affected by their viability (37, 38) highlighting the advantages of these methods, as they allow for conclusions about variations in inflammatory potential based on the microbial composition (2, 39, 40). However, active sampling methods that rely solely on culture-based methods have some limitations such as only allowing the evaluation of culturable microorganisms which can cause an underestimation of the microbial load due to the potential cell damage caused by the high velocity of the airflow of this equipment (41, 42). Also, the fact that indoor air is not homogeneous in space or time makes this type of sample a peculiar one since it can change based on the type and intensity of the activity that is being developed in each specific sampling site (1). To overcome those limitations, passive sampling methods are a reliable choice since they allow a complete assessment by not only collecting contamination over a longer period covering all the mentioned air variations (1, 43), but by also facilitating the combination of different approaches such as culture-based methods and molecular tools (44), as well as to analyse metabolites such as mycotoxins, more straightforward, since there are methods with a longer sampling period such as EDCs. Therefore, it is clear that sampling protocols should include more than one type of sampling method, specifically, comprising active and passive sampling methods (45, 46).

As previously stated, it is important to consider the impact of seasonal variations and the influence of parameters that depend on the season (such as temperature and relative humidity of indoor and outdoor air) when analyzing indoor microbial concentrations (3). These parameters can also be influenced by the type of ventilation present in each carpentry. Although all facilities were equipped with woodworking machines that included localized ventilation systems, only half of the carpentries (3 out of 6) had both natural ventilation (operable windows or doors/gates opened during the sampling campaigns) and general mechanical ventilation systems operating at the building level.

In this study, similar to what was found in previous studies carried out in carpentries (1), *Aspergillus* sp., *Penicillium* sp., and *Cladosporium* sp. were the most prevalent genera among all sampling methods in both seasons in MEA and DG18, with higher prevalence in the warm season. These results corroborate the impact of seasonality on microbial contamination since temperature fluctuations and precipitation patterns can directly influence microbial (fungal and bacterial) growth, survival, distribution, and susceptibility (47) posing serious challenges and a critical public health concern, potentially heightening the risks of exposure and infection for workers in occupational settings.

The WHO recently developed the first fungal priority pathogens list to systematically prioritize fungal pathogens, considering the need to strengthen the global response to fungal infections and antifungal resistance (12). Our group has been aligned with this purpose of global surveillance of antifungal resistance since 2016, through systematic screening of antifungal resistance in different occupational and other environments, to raise awareness and foster appropriate public health policy interventions (39, 40). This study in Portuguese carpentries was prompted by a study carried out in Norwegian sawmills by Viegas et al. (39), and enabled the identification of *Fusarium* sp. and *Mucor* sp. (among other species) with reduced susceptibility to azoles at tested conditions. *Mucor* sp. (order Mucorales) was observed in three different azoles in both seasons, and *Fusarium* sp. in itraconazole in the cold season.

*Fusarium* spp. and Mucorales are both ranked by the WHO in the high-risk priority group. Mucorales is a large group of different fungal genera that are globally distributed and cause a wide spectrum of infection termed mucormycosis. Mucormycosis particularly affect the immunocompromised, individuals with poorly controlled diabetes mellitus and those who have experienced trauma, particularly skin and soft-tissue injuries, whereas invasive mucormycosis is a life-threatening disease with high mortality (48). Mucormycosis was prioritized by India in 2021 under the notifiable disease category, as a result of the world’s largest outbreak thus far, associated with the COVID-19 pandemic. *Fusarium* sp. belong to a large genus of globally distributed filamentous fungi which are found in nature and can infect humans to cause fusariosis. Invasive fusariosis is a life-threatening disease, with mortality ranging from 43 to 67% (12).

These pathogens are also highly antifungal resistant ranked top in terms of research need. Antifungal resistance is difficult to determine, as clinical breakpoints have not been established. Mucorales are inherently resistant to fluconazole, voriconazole and echinocandins, with MICs for azoles generally higher for *Mucor* sp. compared with other species form Mucorales order (such as *Rhizopus* sp.). Regarding *Fusarium* sp., it seems to be inherently resistant to most antifungal agents, with generally lower susceptibility to azoles than to amphotericin B (12). In this study, we used the EUCAST clinical breakpoints for *Aspergillus fumigatus*, using an adapted method to screen fungal susceptibility, as previously described (49, 50). In order to confirm azole-resistance phenotypes, further microdilution tests must be carried out.

Regarding *Aspergillus* section *Fumigati*, it is a ubiquitous environmental fungi that can be inhaled and infect humans and cause aspergillosis (ranging from an allergic reaction to acute invasive aspergillosis). It is ranked in the critical group by WHO, due to the fact that azole-resistant invasive aspergillosis is a life-threatening disease with very high mortality. Although aspergillosis is preventable (by antifungal prophylaxis in high-risk groups), emerging resistance to azoles is concerning. The widespread use of azole fungicides in agriculture to prevent crop losses is contributing to the rising rates of resistant aspergillosis in humans (51). In this study, *Aspergillus* section *Fumigati* was observed in MEA, DG18 and SDA, but not in azole-supplemented media. This may be due to the absence of resistance to azoles in the collected *Aspergillus* or may be underestimated due to the presence of fast-growing species (e.g., order Mucorales) causing competition for nutrients and inhibiting the growth of other fungal species such as *Aspergillus* section *Fumigati* (52). In fact, that is considered a limitation of culture-based methods, although they remain widely used for fungal identification. The growth rate and environmental requirements of different fungal species can influence outcomes in mixed cultures. For example, and as previously mentioned, a fast-growing species may dominate, potentially leading to chemical competition that inhibits the growth of slower-growing species (6, 22). This imbalance can reduce the accuracy of results. Consequently, integrating culture-based methods with molecular tools offers a more robust approach. Molecular tools provide advantages such as higher accuracy, speed, exceptional analytical sensitivity, and the ability to detect and identify dead or dormant microorganisms, as well as toxigenic strains of specific fungal species (22, 53). Indeed, it was possible to detect *Aspergillus* section *Fumigati* in samples where it was not possible to observe. Thus, the complementary use of both methods is strongly recommended, as demonstrated in previous studies (22, 46, 54). Additionally, it is important to highlight that, there is no safe level of exposure to pathogenic microorganisms, therefore, their presence should be null (55).

Mycotoxins are fungal metabolites that can remain in the environment for longer periods than viable fungi due to their ability to sustain severe environmental conditions (12, 56). The primary concern with mycotoxins lies in their widespread presence and the toxic effects they exert on humans and animals, as they have been identified as cytotoxic, nephrotoxic, hepatotoxic, teratogenic, immunosuppressive, mutagenic, and even carcinogenic. However, despite humans and animals being exposed to multiple mycotoxins simultaneously, risk assessments and regulatory measures have typically focused on individual mycotoxins rather than their combined effects (57). Six different mycotoxins were found in the assessed carpentries, namely fumonisin B1, B2, and B3, griseofulvin, mycophenolic acid and neosolaniol, with three of them (fumonisin B1, griseofulvin and neosolaniol) in both seasons. These results suggest that seasonal variations impact mycotoxins production, as supported by Leggieri and colleagues (58). This is due to the influence of seasonal variations on fungal species prevalence, thereby altering the profile of produced mycotoxins and exposure risks in each season.

To the author’s knowledge, this is the first report of mycotoxins in this occupational setting, however, tremorgenic mycotoxins, which are a specific subgroup of mycotoxins that are neurotoxic compounds capable of causing neurological symptoms in animals and humans, were reported in 2 studies developed in sawmills from Sweden (59, 60) as well as the impact of seasonal variation on them (61, 62). To prevent the presence of these metabolites, it is crucial to prevent the presence of fungi in the first place (63) with some measures such as cleaning and disinfecting surfaces frequently, control humidity levels, improve ventilation, fix moisture issues to stop fungal growth, store materials in dry, cool conditions, inspect them regularly for contamination, and clean surfaces and dust frequently (64, 65).

Different toxicity profiles were identified (via the MTT assay) in passive samples collected from carpentries, with differences between seasons and sample type. The MTT test has a high sensitivity relative to fungi and their secondary metabolites and is a fast tool to assess their cytotoxicity (66). The used cell lines were chosen to mimic the most probable routes of exposure to microbes and their toxins/metabolites: A549 human lung epithelial cells as a model for exposure by inhalation; and swine kidney cells as a model for exposure by ingestion. Settled dust and filters accounted for the main differences observed among seasons for both cell lines. These results corroborate the influence of sampling methods in the contamination load and type and their toxicity, as previously stated (61, 62). Several contaminants of biological origin are known to be involved in cellular death, including multiple mycotoxin-induced toxicities (67). Nevertheless, the observed cytotoxicity may also be related to chemicals or particulate matter speciation that were not assessed in this study.

The cytotoxicity assessment developed in environmental samples comprises the toxicity of the mixture of contaminants present in each sample, which can be different from the sum of toxicity of each contaminant individually due to synergism (68). Exposing cells to the entire sample composed of mixed contaminants simulates the real exposure situation since lung cells are exposed directly to the pollutants (69), allowing us to understand the correlation between all environmental contaminants (68). In order to develop a more accurate cytotoxicity assessment in future studies, dust size identification and particles´ speciation should be developed aiming to identify which particles can effectively enter and be harmful to the human respiratory system, allowing its individual cytotoxicity assessment.

Besides microorganisms and its metabolites, woodworkers are exposed to several other agents that can potentially be health-harming, being wood dust one of those agents. As previously mentioned, the particles of solid matter that form wood dust are also known as PM (70) and the size of the PM influences its deposition in the respiratory system, and may cause several adverse health effects (71), such as inflammatory responses that are more strongly triggered by coarse particulate matter than fine and ultrafine particulate matter (72, 73). According to our study, PM contamination was higher for PM10 in most sampling sites, followed by PM5, indicating that wood dust may cause illness by affecting the upper and bigger airways and can also reach the gas exchange zone of the lungs (11). PM can also act as a carrier of microorganisms and their metabolites for the respiratory system since they tend to aggregate with particles of different sizes depending on the source, species, relative humidity, and aerosolization mechanism (28, 74–76) highlighting the importance its evaluation in this study.

The comparison between seasons showed some relevant information regarding the fungal contamination and the particulate matter in each season. The cold season presented the highest prevalence of fungal contamination in all active sampling methods and in most passive sampling methods which can be explained by environmental factors that influence fungal growth such as higher humidity and temperature (77).

The identification of *Aspergillus* sp. with high prevalence on DG18 incubated at 37°C by active and passive sampling raises concerns about workers’ potential exposure to potential pathogenic fungi such as *Aspergillus fumigatus* which integrates the WHO critical group.

There are some limitations in this study such as the lack of dust size identification in personal air samples, as well as the fact that most sampling methods were applied on a single day in each season. Additionally, PM monitoring was conducted only once per season at each sampling site, with measurements lasting only 5 min, which may be too short to capture fluctuations in PM concentrations during the day.

## 5 Conclusion

This study’s integrated approach, which included diverse sampling methods, laboratory assays, and consideration of seasonality, provided a comprehensive analysis of environmental factors and supports targeted risk assessment and management strategies. Overall, some concerns were raised, such as: (a) the fungal species with clinical relevance found in this study, the possibility of fungal exposure through inhalation able to promote respiratory diseases; (b) the high prevalence of *Aspergillus* sp. incubated at 37°C which corroborates their pathogenic potential in both seasons; (c) azole-resistant species, such as *Mucor* sp. and *Fusarium* sp., were found, raising concerns due to their potential to cause invasive fungal infections with severe clinical outcomes; (d) detection of several mycotoxins for the first time in this setting and with differences between seasons; (e) high levels of cytotoxicity in human lung epithelial cells, suggesting high toxicity of the co-exposure via inhalation; (f) seasonal variations effect on microbial contamination (species diversity, prevalence, frequency and resistance), highlighting the role of environmental conditions (humidity and temperature) in influencing microbial growth, survival, and pathogenicity.

Based on the data obtained, future research, aiming to overcome the limitations of this study, could focus on: (a) conducting a more targeted evaluation of size-specific particles, which may provide a more accurate understanding of the potential health effects related to dust exposure; (b) developing a longitudinal study within this occupational setting to enable a longer sampling campaign, addressing the limitations of cross-sectional studies while offering valuable insights into exposure determinants and their changes over time; (c) conducting a study that correlates the exposure data with health effects data in these workers population; and (d) conducting a study that correlates environmental variables and microbial contamination levels.

## Data Availability

The original contributions presented in the study are included in the article/Supplementary material, further inquiries can be directed to the corresponding author.
