# Supplementary material for: Boosting knowledge on occupational exposure to microbial contamination in Portuguese carpentries
Source: Front Public Health. 2025 Jun 6;13:1574881. doi: 10.3389/fpubh.2025.1574881 (PMC12180304; doi:10.3389/fpubh.2025.1574881)
Supplement: Supplementary file 1 [file Data_Sheet_1.docx]

Supplementary Material

Table S1 – Walkthrough Survey Carpentries

|  | **Yes** | **No** | **N/A** | **Answer/Comments** |
| --- | --- | --- | --- | --- |
| 1. **General Information** | | | | |
| - 1. Building age |  |  |  |  |
| - 1. Start of operations |  |  |  |  |
| - 1. Building area |  |  |  |  |
| - 1. Number of workers |  |  |  |  |
| - 1. How many workstations are there? |  |  |  |  |
| - 1. What job is performed in each workstation? |  |  |  |  |
| - 1. What machines are in each workstation? |  |  |  |  |
| - 1. What is the final product? |  |  |  |  |
| - 1. Is there a storage area for the wood? |  |  |  |  |
| - 1. Does the building have natural ventilation? |  |  |  |  |
| - 1. Does the building have mechanical ventilation? If so, is it general or localised? |  |  |  |  |
| 1. **Wood** (1–4) | | | | |
| - 1. What types of wood are used? |  |  |  |  |
| - 1. Where does the wood come from? |  |  |  |  |
| - 1. Do you use solvents or varnishes? If so, which ones? |  |  |  |  |
| - 1. Do you use antifungal agents to treat the wood? If so, which ones? |  |  |  |  |
| 1. **Personal Protective Equipment** (5) | | | | |
| - 1. What PPE workers use? |  |  |  |  |
| - 1. How often do they replace them? |  |  |  |  |
| - 1. Do they have a work uniform? |  |  |  |  |
| - 1. Do they have individualised lockers? |  |  |  |  |
| - 1. Do they have divided lockers (civilian clothes/work uniform)? |  |  |  |  |
| 1. **Cleaning and Disinfecting Procedures** (6,7) | | | | |
| - 1. Do you have procedures for cleaning and sanitizing work areas? If so, which ones? |  |  |  |  |
| - 1. How often do you clean and sanitise work areas? |  |  |  |  |
| - 1. What products are used? |  |  |  |  |
| 1. **Waste Management (Wood Shavings)** (1–4) | | | | |
| - 1. What is the destination of the shavings? |  |  |  |  |
| - 1. What is the destination of the sawdust? |  |  |  |  |
| - 1. How are the shavings packaged? |  |  |  |  |
| - 1. How is the sawdust packaged? |  |  |  |  |
| - 1. What is the sawdust extraction process? |  |  |  |  |
| - 1. What is the process of channelling the chips to their final destination? |  |  |  |  |
| - 1. What is the process for transporting sawdust to its final destination? |  |  |  |  |
| 1. **Basic occupational health and safety activities** | | | | |
| - 1. Are there organised occupational health and safety services? |  |  |  |  |
| - 1. What type of services are used? Internal, external or joint? |  |  |  |  |
| - 1. Are there occupational health and safety technicians with a valid professional qualification who are suitably qualified to meet the company's needs? |  |  |  |  |
| - 1. Is there an employer's representative in the case of external OSH services? |  |  |  |  |
| 1. **Training for workers** | | | | |
| - 1. Are workers properly informed of the risks to which they are exposed? |  |  |  |  |
| 1. **Thermal conditions** | | | | |
| - 1. Do most occupants complain of thermal discomfort? |  |  |  |  |
| - 1. Are the temperature (18<RefTºC<24) and relative humidity (50<RH%<70) suitable for the task at hand? |  |  |  |  |
| - 1. Are there permanent sources of heat or cold that produce thermal discomfort? |  |  |  |  |
| - 1. Are there structural components with characteristics that minimise the impact of external temperatures? |  |  |  |  |
| - 1. Is there air conditioning equipment (heating and cooling)? Is it sufficient and in good working order? |  |  |  |  |
| 1. **Air Quality** | | | | |
| - 1. Are there any complaints from the majority of occupants (e.g. respiratory and/or eye irritation, headaches, difficulty concentrating, itchy and/or dry skin, dry cough)? |  |  |  |  |
| - 1. Is there damp and/or mould on structural elements and/or furniture? |  |  |  |  |
| - 1. Is the workspace overcrowded? |  |  |  |  |
| - 1. Are the means of natural and/or mechanical ventilation insufficient or non-existent? |  |  |  |  |
| - 1. Are there insufficient or no ventilation practices in the premises? |  |  |  |  |

1. Derkyi N. Azoles for Renewable Energy Development and Wood Treatment. In 2020.

2. Kuznetsov YI, Kazansky LP. Physicochemical aspects of metal protection by azoles as corrosion inhibitors. Russ Chem Rev. 2008 Mar 31;77(3):219.

3. Sana S, Reddy VG, Bhandari S, Reddy TS, Tokala R, Sakla AP, et al. Exploration of carbamide derived pyrimidine-thioindole conjugates as potential VEGFR-2 inhibitors with anti-angiogenesis effect. Eur J Med Chem. 2020 Aug 15;200:112457.

4. Straumfors A, Corbin M, McLean D, ‘t Mannetje A, Olsen R, Afanou A, et al. Exposure Determinants of Wood Dust, Microbial Components, Resin Acids and Terpenes in the Saw- and Planer Mill Industry. Annals of Work Exposures and Health. 2020 Mar 10;64(3):282–96.

5. Viegas C, Dias M, Almeida B, Aranha Caetano L, Carolino E, Quintal Gomes A, et al. Are workers from waste sorting industry really protected by wearing Filtering Respiratory Protective Devices? The gap between the myth and reality. Waste Manag. 2020 Feb 1;102:856–67.

6. Dias M, Sousa P, Viegas C. Occupational Exposure to Bioburden in Portuguese Ambulances. In: Arezes PM, Baptista JS, Carneiro P, Castelo Branco J, Costa N, Duarte J, et al., editors. Occupational and Environmental Safety and Health III [Internet]. Cham: Springer International Publishing; 2022 [cited 2023 Feb 27]. p. 167–73. (Studies in Systems, Decision and Control). Available from: https://doi.org/10.1007/978-3-030-89617-1_15

7. Viegas C, Twarużek M, Dias M, Almeida B, Carolino E, Kosicki R, et al. Assessment of the microbial contamination of mechanical protection gloves used on waste sorting industry: A contribution for the risk characterization. Environ Res. 2020 Oct;189:109881.

8. Hanelt M, Gareis M, Kollarczik B. Cytotoxicity of mycotoxins evaluated by the MTT-cell culture assay. Mycopathologia. 1994 Dec;128(3):167–74.

9. Gniadek A, Krzyściak P, Twarużek M, Macura AB. Occurrence of fungi and cytotoxicity of the species: Aspergillus ochraceus, Aspergillus niger and Aspergillus flavus isolated from the air of hospital wards. Int J Occup Med Environ Health. 2017 Mar 30;30(2):231–9.

10. Viegas C, Twarużek M, Almeida B, Dias M, Ribeiro E, Carolino E, et al. Cytotoxicity of Aspergillus Section Fumigati Isolated from Health Care Environments. Journal of Fungi. 2021 Oct;7(10):839.

Table S2 – Sampling details from active and passive sampling methods

|  | **Sampling Method** | **Sampling Strategy Details** |
| --- | --- | --- |
| **Active Sampling Methods** | MAS-100 air tester | 100 L/min for 2 min. |
|  | Andersen six-stage cascade impactor | 28.3 L/min, for 9 min in each culture medium. |
|  | Coriolis μ air sampler | 300 L/min for 2 min with air samples being pumped into vials with 10 ml sterile phosphate-buffered saline (PBS - pH 7.4) with 0.05% Triton X100. |
|  | SKC Button Aerosol Sampler connected to a SKC air sampling pump | 4L/min for 2 hours in 2 workers. |
| **Passive Sampling Methods** | Electrostatic dust collectors (EDC) | Placed in the sampling areas 1.5 m above the ground for 30 days. |
|  | Floor surface swabs | Swabbed using a 20 cm^2^ square stencil (disinfected between each sampling with a 70% alcohol solution). |
|  | E-cloths (EDCP) | Placed in three workers from different working areas with safety pins, during the entire work shift. |
|  | Settled dust and filter | Settled dust was vacuumed with a common vacuum cleaner (and respective filter). The dust was analysed as a composite sample of the whole store since the amount of dust was reduced due to the cleaning practices. |
|  | Filtering respiratory protection devices (FRPD) | FRPD (models N95, chemical mask with 6051 Dust Mask Filter and surgical masks were collected at the end of the work shift, but it is important to highlight that all of them had several days of use (1 to 4 days each). |
|  | Mechanical protection gloves (MPG) | MPG (model EN 388) was collected at the end of the work shift, but it is important to highlight that it had already 3 days of use. |

| **Sampling Method** | **Extraction for characterization of viable microbiota** |
| --- | --- |
| Filter from personal air sampler | Washed with 10 mL of 0.1% Tween 80 saline (0.9% NaCl) solution (250 rpm, 30 min). |
| Electrostatic dust collectors (EDC) | Washed with 10 mL of 0.1% Tween 80 saline (0.9% NaCl) solution (250 rpm, 30 min). |
| Floor surface swabs | Washed with 1 mL of 0.1% Tween 80 saline (0.9% NaCl) solution (250 rpm, 30 min). |
| E-cloths (EDCP) | Washed with 10 mL of 0.1% Tween 80 saline (0.9% NaCl) solution (250 rpm, 30 min). |
| Settled dust | Washed with 9.1 mL/g of 0.1% Tween 80 saline (0.9% NaCl) solution (250 rpm, 30 min). |
| Filter from vacuumed dust | Washed with 10 mL of 0.1% Tween 80 saline (0.9% NaCl) solution (250 rpm, 30 min). |
| Filtering respiratory protection devices (FRPD) | Washed with 10 mL of 0.1% Tween 80 saline (0.9% NaCl) solution (250 rpm, 30 min). |
| Mechanical protection gloves (MPG) | Washed with 10 mL of 0.1% Tween 80 saline (0.9% NaCl) solution (250 rpm, 30 min). |
|  |  |

Table S3 – Extraction details for characterization of viable microbiota

**Table S4 –** Sequence of primers and TaqMan probes used for Real-Time PCR

| ***Species targeted**** | | **Sequences** | | **Reference** | |
| --- | --- | --- | --- | --- | --- |
| ***Fumigati*** |  | |  | |  |
| Forward Primer | 5‘-CGCGTCCGGTCCTCG-3‘ | |  | |  |
| Reverse Primer | 5‘-TTAGAAAAATAAAGTTGGGTGTCGG -3‘ | | (Cruz-Perez et al. 2001) | |  |
| Probe | 5‘-TGTCACCTGCTCTGTAGGCCCG -3‘ | |  | |  |
| ***Circumdati*** | |  | |  | |
| Forward Primer | | 5‘-CGGGTCTAATGCAGCTCCAA-3‘ | |  | |
| Reverse Primer | | 5‘-CGGGCACCAATCCTTTCA-3‘ | | (Viegas et al. 2017b) | |
| Probe | | 5‘-CGTCAATAAGCGCTTTT-3‘ | |  | |
| ***Nidulantes***  Forward Primer  Reverse Primer  Probe | | 5’ – CGGCGGGGAGCCCT-3’  5’ – CCATTGTTGAAAGTTTTGACTGATcTTA-3’  5’ – AGACTGCATCACTCTCAGGCATGAAGTTCAG-3’ | | (EPA 2017) | |

*Reactions included 1 × iQ Supermix (Bio-Rad, Portugal), 0.5 μM of each primer, and 0.375 μM of TaqMan probe in a total volume of 20 μl.

**Table S5 –** Sequence of primers and TaqMan probes used for Real Time PCR

| **Amplification* step** | Temperature | Time |
| --- | --- | --- |
| *Denaturation* | 95ºC | 30s |
| Annealing | 52ºC | 30s |
| Extension | 72ºC | 30s |

* The amplification included 40 cycles

**Table S6** – Extraction details for mycotoxins detection

| **Sampling Method** | **Mycotoxins analysis** |
| --- | --- |
| Electrostatic dust collectors (EDC) | Raw extracts were diluted with an equal amount of water, mixed, centrifuged, and then injected into the LCMS/MS system for analysis. |
| Floor surface swabs |  |
| E-cloths (EDCP) |  |
| Settled dust |  |
| Filtering respiratory protection devices (FRPD) |  |
| Mechanical protection gloves (MPG) |  |
| Filter from vacuumed dust | Filter samples (0.2 g) were subjected to extraction with 2.0 ml of ACN: H2O: AcOH (79:20:1) for 60 minutes. |
| Filter from personal air sampler |  |
| Coriolis µ air sampler | For air samples (100 μl), a direct dilution of 1:7 (v/v) was conducted using a mixture of water and extraction solvent (acetonitrile (ACN): water (H2O): acetic acid (AcOH) (79:20:1)). |

**Table S7** – The Limits of Detection (LOD) for each mycotoxin

| **Mycotoxin** | **Type of Sample** | |
| --- | --- | --- |
|  | **LOD for Coriolis** | **LOD for Filters, EDCP, EDC, Settled Dust, Gloves and Masks** |
| **15-Acetyldeoxynivalenol** | 7,8 | 8,1 |
| **3-Acetyldeoxynivalenol** | 5,4 | 5,7 |
| **Aflatoxin B1** | 0,6 | 0,5 |
| **Aflatoxin B2** | 0,5 | 0,5 |
| **Aflatoxin G1** | 0,5 | 0,5 |
| **Aflatoxin G2** | 0,9 | 0,7 |
| **Aflatoxin M1** | 0,9 | 0,8 |
| **aZearalanol** | 1,3 | 1,3 |
| **aZearalenol** | 0,7 | 1,0 |
| **bZearalanol** | 2,7 | 3,3 |
| **bZearalenol** | 1,8 | 1,9 |
| **Deepoxydeoxynivalenol** | 3,3 | 4,3 |
| **Deoxynivalenol** | 2,8 | 3,5 |
| **Deoxynivalenol-3-Glucoside** | 4,2 | 4,2 |
| **Diacetoxyscirpenol** | 1,9 | 2,3 |
| **Fumonisin B1** | 12,7 | 12,2 |
| **Fumonisin B2** | 7,2 | 7,5 |
| **Fumonisin B3** | 9,0 | 8,4 |
| **Fusarenon X** | 3,5 | 4,5 |
| **Gliotoxin** | 4,0 | 4,2 |
| **Griseofulvin** | 2,8 | 2,6 |
| **HT2** | 3,4 | 3,6 |
| **Mevinolin** | 2,4 | 2,2 |
| **Moniliformin** | 3,2 | 2,7 |
| **Monoacetoxyscirpenol** | 2,8 | 3,1 |
| **Mycophenolic acid** | 3,3 | 3,5 |
| **Neosolaniol** | 1,9 | 3,1 |
| **Nivalenol** | 4,3 | 4,3 |
| **Ochratoxin A** | 0,8 | 0,6 |
| **Ochratoxin B** | 1,3 | 1,5 |
| **Patulin** | 7,5 | 8,3 |
| **Roquefortine C** | 3,5 | 4,1 |
| **Sterigmatocystin** | 0,7 | 0,7 |
| **T2** | 1,0 | 0,9 |
| **T2 Tetraol** | 8,8 | 8,0 |
| **T2 Triol** | 6,0 | 5,5 |
| **Zearalanon** | 1,3 | 1,2 |
| **Zearalenon** | 0,5 | 0,8 |

**Methods Supplement S1 –** Cytotoxicity Evaluation

Cells were maintained in Minimum Essential Medium Eagle (MEM) (Sigma-Aldrich, St. Louis, MI, USA) supplemented with an antibiotic solution (stock solution: 10,000 units of penicillin and 10 mg of streptomycin per mL in 0.9% NaCl (Sigma Aldrich)), and 5% fetal calf serum (Sigma-Aldrich) in a CO_2_-incubator (CB, BINDER GmbH, Tuttlingen, Germany) (5% CO_2_, 37˚C, 98% humidity). Cells were detached from the bottom of the culture vessel using 0.25% (w/v) Trypsin 0.53 mM EDTA and then suspended in the culture medium. Their number was determined using a cell counter (Scepter™ 2.0 Cell Counter, Merck Millipore, Burlington, MA, USA). Subsequently, 2.5 x 10^5^ cells were seeded per well of a 96-well microtiter plate. Cell suspensions (100 μl) were then incubated with the test samples in a 96-well plate for 48 h at 5% CO_2_, 37 ◦C, and a humid atmosphere. Then, MTT (3-(4,5-dimethylthiazol-2-yl)-2,5-diphenyltetrazolium bromide) solution (20 µL) was added, and plates were incubated for another 4 h. Subsequently, the supernatant was removed, and 100 µL dimethyl sulfoxide (DMSO) was added to each well. The cytotoxicity level was measured at 510 nm (ELISA LEDETECT 96, biomed Dr. Wieser GmbH; MikroWin 2013SC software). The lowest concentration, dropping absorption to <50% of cell metabolic activity (IC50), was considered the threshold toxicity level. Semi-quantitative scale for cytotoxicity grading was adopted: low cytotoxicity (+) with IC50 values ranging from 31.251 cm2 /ml to 7.813 cm2 /ml; medium cytotoxicity (++) with IC50 values ranging from 3.906 cm2 /ml to 0.977 cm2 /ml; high cytotoxicity (+++) with IC50 values ranging from 0.488 cm2 /ml to 0.061 cm2 /ml. The absence of cytotoxicity was considered when the extract concentration at 31.251 cm2 /ml failed to inhibit the growth of the swine kidney and A549 cells (1–3).

1. Hanelt M, Gareis M, Kollarczik B. Cytotoxicity of mycotoxins evaluated by the MTT-cell culture assay. Mycopathologia. 1994 Dec;128(3):167–74.

2. Gniadek A, Krzyściak P, Twarużek M, Macura AB. Occurrence of fungi and cytotoxicity of the species: Aspergillus ochraceus, Aspergillus niger and Aspergillus flavus isolated from the air of hospital wards. Int J Occup Med Environ Health. 2017 Mar 30;30(2):231–9.

3. Viegas C, Twarużek M, Almeida B, Dias M, Ribeiro E, Carolino E, et al. Cytotoxicity of Aspergillus Section Fumigati Isolated from Health Care Environments. Journal of Fungi. 2021 Oct;7(10):839.

Table S8 – Sampling details from particulate matter monitoring

| **Equipment** | **Monitoring Strategy Details** |
| --- | --- |
| Lighthouse Handheld Particle Counter | 2.83 L/min for 7 minutes (2 minutes to stabilize the equipment and 5 minutes for the sampling). |

Table S9 - Characterization of the 6 carpentries sampled

| **Carpentry** | **Building age (years)** | **Sawmill area (m2)** | **Type of ventilation** | **Wood** | | **Number of workers** | **Cleaning frequency (per day)** | **Cold Season** | | | | | | | | | **Warm Season** | | | | | | | | | |
| --- | --- | --- | --- | --- | --- | --- | --- | --- | --- | --- | --- | --- | --- | --- | --- | --- | --- | --- | --- | --- | --- | --- | --- | --- | --- | --- |
|  |  |  |  |  |  |  |  | **Active sampling methods** | | | | | **Passive sampling methods** | | | | **Active sampling methods** | | | | | **Passive sampling methods** | | | | |
|  |  |  |  | **Type** | **Origin** |  |  | **MAS-100** | **Andersen six stage** | **Button Sampler** | **Coriolis µ Air Sampler** | **Lighthouse** | **Surface swabs** | **EDC** | **EDCP** | **Settled dust + Filters** | **MAS-100** | **Andersen six stage** | **Button Sampler** | **Coriolis µ Air Sampler** | **Lighthouse** | **Surface swabs** | **EDC** | **EDCP** | **Settled dust + Filters** | **MPG** |
| A | 28 | 250 | Natural | Particleboard, MDF, Black pine, plywood | Portugal | 6 | 2 | 16 | 18 | 2 | 3 | 4 | 3 | 3 | 2 | 1 | 16 | 18 | 2 | 3 | 4 | 3 | 3 | 2 | 1 | 0 |
| B | 28 | 911 | Natural and artificial | Beech, oak, wenge, mahogany, pine, white wood,tola, sucupira wood, riga, cherry, jatoba, garapa, kambala, afzelia | Portugal and exotic countries | 21 | UNK | 16 | 18 | 2 | 3 | 4 | 3 | 3 | 2 | 3 | 16 | 18 | 2 | 3 | 4 | 3 | 3 | 2 | 3 | 0 |
| C | 30 | UK | Natural and artificial | UKN | Portugal and International | 6 | 1/week | 16 | 18 | 2 | 3 | 4 | 4 | 4 | 2 | 3 | 16 | 18 | 2 | 3 | 4 | 4 | 4 | 2 | 3 | 0 |
| D | 40 | 80 | Natural and artificial | Derivatives, pine, magno, cambola | Portugal and African continent | 2 | 1/week | 12 | 12 | 1 | 2 | 3 | 2 | 2 | 1 | 2 | 12 | 12 | 1 | 2 | 3 | 2 | 2 | 2 | 3 | 0 |
| E | 30 | 600 | Natural | Particleboard, MDF, plywood | Portugal and France | 7 | UNK | 12 | 12 | 1 | 2 | 3 | 2 | 2 | 1 | 2 | 12 | 12 | 2 | 2 | 3 | 2 | 2 | 1 | 4 | 1 |
| F | 47 | 250 | Natural | Tola, oak, pine, MDF, chipboard | International | 8 | 1-2/week | 12 | 12 | 2 | 2 | 3 | 2 | 2 | 2 | 2 | 12 | 12 | 2 | 2 | 3 | 2 | 2 | 2 | 2 | 0 |
| **Total** | | | | | | | | **84** | **90** | **10** | **15** | **21** | **16** | **16** | **10** | **13** | **84** | **90** | **11** | **15** | **21** | **16** | **16** | **11** | **16** | **1** |

**Table S10 -** Fungal and bacterial load (CFU/m^3^) in active sampling methods per sampling site in each season

|  | **Warm Season** | | | | | **Cold Season** | | | | |
| --- | --- | --- | --- | --- | --- | --- | --- | --- | --- | --- |
|  | **MEA** | **DG18** | **DG18 37ºC** | **TSA** | **VRBA** | **MEA** | **DG18** | **DG18 37ºC** | **TSA** | **VRBA** |
| **Button Samplers (CFU/m^3^)** | | | | | | | | | | |
| **BZ** | 4.5 | 21.9 | 0 | 19.1 | 24.7 | 5.2 | 10.4 | 1.0 | 97.9 | 9.4 |
| **MZ** | 7.6 | 4.9 | 0 | 2.8 | 12.5 | 8.9 | 25 | 0 | 67.7 | 341.7 |
| **BZ/MZ** | - | - | - | - | - | 29.2 | 14.6 | 1.0 | 76.0 | 4.2 |
| **MAS-100 (CFU/m^3^)** | | | | | | | | | | |
| **BZ** | 606 | 755 | - | 928 | 708 | 310 | 984 | - | 1574 | 20 |
| **MZ** | 1013 | 458 | - | 762 | 386 | 277 | 1014 | - | 816 | 106 |
| **W** | 95 | 257.5 | - | 800 | 15 | 325 | 1992.5 | - | 805 | 15 |
| **O** | 42.5 | 60 | - | 640 | 0 | 192.5 | 195 | - | 950 | 0 |
| **E** | 593.3 | 600.8 | - | 273.3 | 585 | 605.8 | 940 | - | 1270 | 16.7 |

**Table S11 -** Fungal and bacterial contamination in passive sampling methods per sampling site in each season

|  | **Warm Season** | | | | | **Cold Season** | | | | | |
| --- | --- | --- | --- | --- | --- | --- | --- | --- | --- | --- | --- |
|  | **MEA** | **DG18** | **DG18 37ºC** | **TSA** | **VRBA** | **MEA** | | **DG18** | **DG18 37ºC** | **TSA** | **VRBA** |
| **EDC (CFU/m^2^/day)** | | | | | | | | | | | |
| **BZ** | 123.7 | 60.8 | 2.1 | 73.4 | 203.4 | 459.2 | | 343.9 | 6.3 | 186.6 | 2.1 |
| **MZ** | 102.8 | 100.7 | 4.2 | 81.8 | 165.7 | 434.1 | | 310.4 | 27.3 | 247.4 | 2.1 |
| **W** | 288.3 | 340.8 | 5.2 | 482.3 | 387.9 | 314.5 | | 115.3 | 0 | 408.9 | 0 |
| **O** | 10.5 | 15.7 | 0 | 173 | 131.1 | 429.9 | | 36.7 | 0 | 36.7 | 0 |
| **EDCP (CFU/m^2^)** | | | | | | | | | | | |
| **BZ** | 3.9 | 2.9 | 1.6 | 36.8 | 5.1 | 1.9 | | 0.9 | 0 | 8.1 | 0.4 |
| **MZ** | 8.1 | 7.7 | 5.2 | 7.9 | 1.7 | 2.2 | | 8.4 | 0.5 | 14.2 | 6.4 |
| **BZ/MZ** | 1.9 | 0 | 0 | 15.4 | 35.2 | 8.5 | | 0.4 | 0 | 11.3 | 0 |
| **Filters (CFU/m^2^)** | | | | | | | | | | | |
| **BZ** | 4416.7 | 7750 | 3250 | 13833.3 | 3500 | 18833.3 | | 10083.3 | 83.3 | 5250 | 83.3 |
| **MZ** | 10416.7 | 27416.7 | 2000 | 1333.3 | 0 | 31000 | | 46583.3 | 500 | 7833.3 | 1500 |
| **W** | 35250 | 94000 | 20250 | 1000 | 0 | 85000 | | 110000 | 1500 | 11750 | 500 |
| **O** | 11000 | 16000 | 0 | 10500 | 0 | 5500 | | 36500 | 0 | 27250 | 6500 |
| **Gloves (CFU/m^2^)** | | | | | | | | | | | |
| **C** | 4500 | 4500 | 0 | 500 | 0 | - | | - | - | - | - |
| **SD (CFU/g)** | | | | | | | | | | | |
| **BZ** | 93.6 | 51.8 | 10 | 64.2 | 12 | 48.4 | | 310.4 | 3.2 | 53.8 | 21 |
| **MZ** | 22.3 | 20.5 | 4.3 | 49.2 | 19.3 | 19.5 | | 66.8 | 3.3 | 48.5 | 4 |
| **BZ/MZ** | - | - | - | - | - | 27 | | 4 | 10 | 56 | 22.50 |
| **Swabs (CFU/m^2^)** | | | | | | | | | | | |
| **BZ** | 70000 | 71666.7 | 56666.7 | 476666.7 | 1666.7 | | 208333.3 | 321666.7 | 0 | 315000 | 75000 |
| **MZ** | 46666.7 | 155000 | 23333.3 | 186666.7 | 21666.7 | 120000 | | 1188333.3 | 0 | 485000 | 13333.3 |
| **W** | 220000 | 190000 | 275000 | 490000 | 1320000 | 100000 | | 490000 | 0 | 640000 | 25000 |
| **O** | 5000 | 30000 | 5000 | 335000 | 1225000 | 20000 | | 115000 | 0 | 280000 | 0 |

**Table S12 -** Molecular detection results from the samples analysed

|  | **Warm Season** | | | | **Cold Season** | | | |
| --- | --- | --- | --- | --- | --- | --- | --- | --- |
| ***Aspergillus* sp.** | **Sample Type** | **Sample** | **CFU/m^2^ (MEA/DG18)** | **Cq** | **Sample Type** | **Sample** | **CFU/m^2^ (MEA/DG18)** | **Cq** |
| *Aspergillus* section *Nidulantes* | Settled Dust | C-SD-W | 0/0 | 34.2 | Settled Dust | C-SD-W | 0/0 | 37.9 |
|  | Settled Dust | B-SD-MZ | 0/0 | 39.8 | Settled Dust | B-SD-MZ | 0/0 | 39.7 |
|  | Filter* | B-F*-MZ | 0/0 | 37.7 | Settled Dust | D-SD-BZ | 0/37 | 36.4 |
|  | Filter* | D-F*-MZ | 0/0 | 35.9 | Settled Dust | D-SD-MZ | 0/0 | 36.3 |
|  |  |  |  |  | Filter* | C-F*-W | 0/1000 | 36.2 |
|  |  |  |  |  | Filter* | D-F*-MZ | 0/0 | 38.6 |
| *Aspergillus* section *Fumigati* | Button Sampler | C-BS-MZ | 0/0 | 37.2 |  |  |  |  |
|  | Button Sampler | F-BS-BZ | 0/0 | 35.2 |  |  |  |  |
|  | Button Sampler | B-BS-BZ | 4.17/0 | 29.9 |  |  |  |  |
|  | Filter* | B-F*-BZ | 7500/0 | 34.3 |  |  |  |  |
|  | Filter* | B-F*-W | 500/0 | 36.5 |  |  |  |  |
|  | Filter* | D-F*-BZ | 0/0 | 38.0 |  |  |  |  |
|  | Filter* | D-F*-MZ | 0/0 | 37.9 |  |  |  |  |
|  | Filter* | E-F*-BZ | 0/0 | 36.6 |  |  |  |  |
|  | Filter* | C-F*-W | 0/0 | 38.3 |  |  |  |  |
|  | Filter* | F-F*-BZ | 0/0 | 37.1 |  |  |  |  |
|  | Filter* | A-F*-MZ | 0/0 | 36.8 |  |  |  |  |
|  | Filter* | A-F*-O | 0/0 | 38.3 |  |  |  |  |
|  | Settled Dust | B-SD-BZ | 0/0 | 34.9 |  |  |  |  |
|  | Settled Dust | B-SD-MZ | 0/0 | 33.8 |  |  |  |  |
|  | Settled Dust | B-SD-W | 0/0 | 34.8 |  |  |  |  |
|  | Settled Dust | D-SD-BZ | 5/0 | 34.4 |  |  |  |  |
|  | Settled Dust | D-SD-MZ | 0/0 | 35.4 |  |  |  |  |
|  | Settled Dust | E-SD-BZ | 0/0 | 36.1 |  |  |  |  |
|  | Settled Dust | E-SD-MZ | 0/0 | 34.2 |  |  |  |  |
|  | Settled Dust | E-SD-A | 0/0 | 35.1 |  |  |  |  |
|  | Settled Dust | E-SD-BZ | 0/0 | 35.3 |  |  |  |  |
|  | Settled Dust | A-SD-MZ | 1/1 | 33.3 |  |  |  |  |
|  | Settled Dust | F-SD-BZ | 0/0 | 35.1 |  |  |  |  |
|  | Settled Dust | F-SD-MZ | 0/0 | 35.5 |  |  |  |  |
|  | Settled Dust | C-SD-W | 0/0 | 35.6 |  |  |  |  |
|  | * Filter from the vacuum cleaner | | | | | | | |


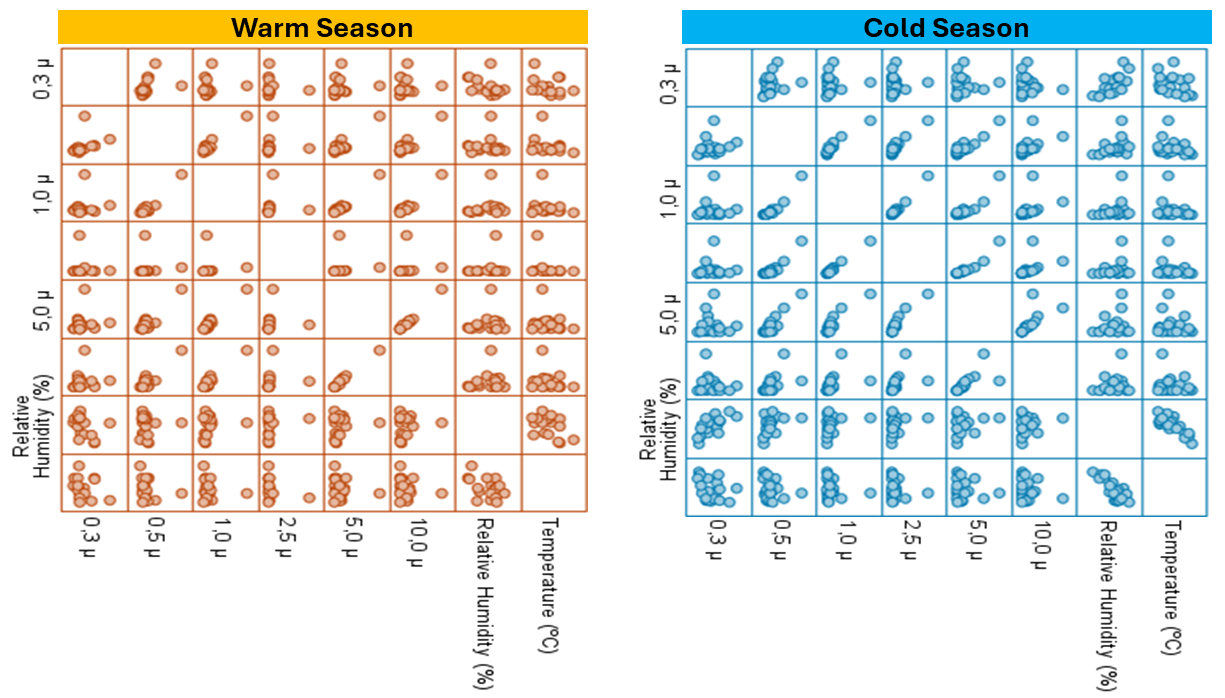


**Figure S1 -** Correlogram of the relationship study between particles of different sizes and environmental conditions in summer and in winter.

**Table S13 -** Study of the relationship between fungal contamination, bacterial contamination, and resistance to azoles between active sampling methods and culture media, in the summer. Spearman correlation coefficient results

|  | | | Andersen | | | | | | | | | | | | | | | | | | | | | | | | MAS 100 | | | | | | | | | Button_Sampler | | | | | |
| --- | --- | --- | --- | --- | --- | --- | --- | --- | --- | --- | --- | --- | --- | --- | --- | --- | --- | --- | --- | --- | --- | --- | --- | --- | --- | --- | --- | --- | --- | --- | --- | --- | --- | --- | --- | --- | --- | --- | --- | --- | --- |
|  |  |  | Fungi | | | | | | | | | | | Bacteria | | | | | | | | | | | | Fungi | | | Bacteria | | | Fungi | | | | | Azole Resistance | | Bacteria | | |
|  |  |  | DG18 Stage 2 | DG18 Stage 3 | DG18 Stage 4 | DG18 Stage 5 | DG18 Stage 6 | DG18 37ºC Stage 1 | DG18 37ºC Stage 2 | DG18 37ºC Stage 3 | DG18 37ºC Stage 4 | DG18 37ºC Stage 5 | DG18_37ºC Stage 6 | TSA Stage 1 | TSA Stage 2 | TSA Stage 3 | TSA Stage 4 | TSA Stage 5 | TSA Stage 6 | VRBA Stage 1 | VRBA Stage 2 | VRBA Stage 3 | VRBA Stage 4 | VRBA Stage 5 | VRBA Stage 6 | MEA | | DG18 | | TSA | VRBA | | MEA | DG18 | DG18 37ºC | | | SDA | | TSA | VRBA |
| Andersen | Fungi | DG18 Stage 1 | 0.147 | 0.237 | 0.139 | **0.548^*^** | **0.825^**^** | -0.239 | -0.346 | -0.244 | -0.189 | 0.072 | -0.169 | -0.147 | -0.176 | -0.188 | -0.165 | 0.132 | 0.072 | 0.267 | 0.246 | 0.364 | 0.368 | 0.129 | 0.328 | **0.535^*^** | | 0.192 | | -0.055 | 0.284 | | -0.121 | -0.017 | -0.322 | | | -0.006 | | -0.050 | -0.302 |
|  |  | DG18 Stage 2 |  | **0.747^**^** | **0.690^**^** | 0.459 | 0.032 | -0.193 | -0.220 | -0.222 | -0.305 | -0.139 | -0.191 | 0.120 | **0.851^**^** | 0.199 | 0.477 | -0.090 | -0.079 | 0.124 | 0.167 | 0.279 | 0.022 | -0.055 | 0.320 | 0.136 | | 0.322 | | -0.042 | -0.006 | | -0.325 | -0.204 | -0.215 | | | -0.369 | | -0.263 | -0.298 |
|  |  | DG18 Stage 3 |  |  | **0.637^*^** | 0.354 | -0.048 | 0.025 | -0.054 | -0.187 | -0.052 | -0.460 | -0.238 | 0.031 | **0.611^*^** | 0.010 | 0.131 | -0.289 | -0.236 | 0.061 | 0.249 | 0.273 | 0.036 | -0.025 | 0.343 | 0.195 | | 0.244 | | -0.155 | 0.039 | | -0.292 | 0.026 | -0.409 | | | -0.399 | | 0.104 | -0.068 |
|  |  | DG18 Stage 4 |  |  |  | **0.674^**^** | -0.084 | -0.166 | -0.230 | -0.216 | -0.353 | -0.211 | -0.309 | **0.532^*^** | **0.597^*^** | 0.186 | 0.406 | 0.335 | 0.425 | **0.621^*^** | **0.768^**^** | **0.794^**^** | **0.606^*^** | 0.470 | **0.797^**^** | **0.545^*^** | | **0.816^**^** | | **0.590^*^** | **0.627^*^** | | -0.037 | -0.155 | -0.155 | | | 0.406 | | 0.040 | -0.523 |
|  |  | DG18 Stage 5 |  |  |  |  | 0.334 | -0.205 | -0.347 | -0.335 | -0.437 | 0.122 | -0.176 | 0.221 | 0.207 | -0.096 | 0.275 | 0.433 | 0.406 | **0.646^**^** | **0.553^*^** | **0.699^**^** | **0.634^*^** | 0.283 | **0.695^**^** | **0.635^*^** | | **0.625^*^** | | 0.465 | **0.523^*^** | | 0.005 | 0.044 | -0.125 | | | 0.409 | | 0.082 | -0.609 |
|  |  | DG18 Stage 6 |  |  |  |  |  | -0.267 | -0.236 | -0.183 | -0.137 | 0.030 | -0.172 | -0.046 | -0.167 | -0.097 | -0.201 | 0.078 | -0.094 | 0.260 | 0.074 | 0.243 | 0.314 | 0.081 | 0.154 | 0.449 | | 0.108 | | -0.065 | 0.190 | | -0.197 | -0.097 | -0.177 | | | -0.022 | | -0.214 | -0.283 |
|  |  | DG18 37ºC Stage 1 |  |  |  |  |  |  | **0.932^**^** | 0.101 | **0.799^**^** | 0.347 | **0.707^**^** | -0.431 | -0.364 | -0.333 | -0.145 | -0.202 | -0.127 | -0.223 | -0.249 | -0.253 | -0.226 | -0.178 | -0.211 | -0.292 | | -0.337 | | -0.123 | -0.216 | | 0.455 | -0.101 | 0.524 | | | -0.154 | | -0.152 | 0.272 |
|  |  | DG18 37ºC Stage 2 |  |  |  |  |  |  |  | 0.109 | .846^**^ | 0.209 | **0.578^*^** | -0.353 | -0.311 | -0.272 | -0.199 | -0.287 | -0.256 | -0.215 | -0.300 | -0.289 | -0.234 | -0.165 | -0.273 | -0.328 | | -0.322 | | -0.119 | -0.230 | | 0.372 | -0.097 | 0.524 | | | -0.203 | | -0.168 | 0.430 |
|  |  | DG18 37ºC Stage 3 |  |  |  |  |  |  |  |  | 0.330 | -0.172 | -0.120 | -0.032 | 0.054 | 0.422 | 0.367 | -0.024 | -0.092 | -0.127 | -0.172 | -0.163 | -0.148 | -0.107 | -0.150 | -0.202 | | -0.230 | | -0.131 | -0.142 | | -0.275 | -0.381 | -0.272 | | | **-0.681^*^** | | -0.296 | 0.377 |
|  |  | DG18 37ºC Stage 4 |  |  |  |  |  |  |  |  |  | 0.026 | 0.454 | -0.484 | -0.388 | -0.041 | -0.222 | -0.237 | -0.403 | -0.200 | -0.302 | -0.256 | -0.201 | -0.258 | -0.198 | -0.221 | | -0.403 | | -0.245 | -0.249 | | 0.288 | -0.242 | 0.341 | | | -0.347 | | -0.282 | 0.420 |
|  |  | DG18 37ºC Stage 5 |  |  |  |  |  |  |  |  |  |  | **0.733^**^** | -0.395 | -0.418 | -0.248 | 0.070 | 0.329 | 0.416 | -0.091 | -0.256 | -0.195 | -0.108 | -0.131 | -0.194 | -0.201 | | -0.214 | | 0.014 | -0.139 | | **0.720^*^** | 0.049 | **0.869^**^** | | | 0.126 | | -0.247 | 0.016 |
|  |  | DG18 37ºC Stage 6 |  |  |  |  |  |  |  |  |  |  |  | **-0.525^*^** | -0.441 | -0.341 | -0.145 | 0.095 | 0.172 | -0.239 | -0.338 | -0.324 | -0.261 | -0.185 | -0.306 | -0.364 | | -0.341 | | -0.131 | -0.251 | | 0.242 | -0.103 | 0.364 | | | -0.201 | | -0.101 | 0.400 |
|  | Bacteria | TSA Stage 1 |  |  |  |  |  |  |  |  |  |  |  |  | 0.415 | 0.328 | 0.402 | 0.406 | 0.405 | **0.570^*^** | **0.672^**^** | **0.616^*^** | **0.583^*^** | **0.537^*^** | **0.546^*^** | 0.463 | | **0.637^*^** | | **0.686^**^** | **0.620^*^** | | -0.319 | -0.381 | -0.331 | | | 0.570 | | -0.085 | -0.461 |
|  |  | TSA Stage 2 |  |  |  |  |  |  |  |  |  |  |  |  |  | 0.460 | **0.642^**^** | -0.098 | -0.140 | 0.104 | 0.148 | 0.200 | -0.031 | -0.081 | 0.243 | 0.068 | | 0.269 | | -0.020 | -0.059 | | -0.468 | -0.179 | -0.309 | | | -0.312 | | -0.193 | -0.231 |
|  |  | TSA Stage 3 |  |  |  |  |  |  |  |  |  |  |  |  |  |  | **0.650^**^** | 0.451 | -0.078 | 0.376 | 0.127 | 0.248 | 0.212 | -0.312 | 0.380 | 0.328 | | 0.042 | | 0.015 | -0.044 | | -0.328 | -0.250 | -0.191 | | | -0.210 | | -0.340 | -0.224 |
|  |  | TSA Stage 4 |  |  |  |  |  |  |  |  |  |  |  |  |  |  |  | 0.424 | 0.207 | 0.307 | 0.165 | 0.254 | 0.150 | -0.200 | 0.346 | 0.220 | | 0.121 | | 0.145 | -0.016 | | -0.355 | -0.470 | -0.152 | | | -0.207 | | -0.516 | -0.411 |
|  |  | TSA Stage 5 |  |  |  |  |  |  |  |  |  |  |  |  |  |  |  |  | **0.707^**^** | **0.799^**^** | **0.577^*^** | **0.662^**^** | **0.750^**^** | 0.234 | **0.694^**^** | **0.656^**^** | | 0.488 | | **0.630^*^** | **0.559^*^** | | -0.104 | -0.214 | -0.148 | | | 0.485 | | -0.061 | -0.324 |
|  |  | TSA Stage 6 |  |  |  |  |  |  |  |  |  |  |  |  |  |  |  |  |  | 0.497 | **0.663^**^** | **0.541^*^** | **0.580^*^** | **0.655^**^** | 0.456 | 0.362 | | **0.630^*^** | | **0.747^**^** | **0.699^**^** | | 0.095 | -0.116 | -0.077 | | | **0.898^**^** | | 0.218 | -0.381 |
|  |  | VRBA Stage 1 |  |  |  |  |  |  |  |  |  |  |  |  |  |  |  |  |  |  | **0.789^**^** | **0.921^**^** | **0.961^**^** | 0.404 | **0.922^**^** | **0.880^**^** | | **0.767^**^** | | **0.743^**^** | **0.765^**^** | | -0.135 | -0.158 | -0.183 | | | 0.504 | | 0.002 | -0.360 |
|  |  | VRBA Stage 2 |  |  |  |  |  |  |  |  |  |  |  |  |  |  |  |  |  |  |  | **0.918^**^** | **0.875^**^** | **0.729^**^** | **0.865^**^** | **0.786^**^** | | **0.900^**^** | | **0.821^**^** | **0.917^**^** | | -0.060 | -0.288 | -0.271 | | | **0.733^*^** | | 0.058 | -0.531 |
|  |  | VRBA Stage 3 |  |  |  |  |  |  |  |  |  |  |  |  |  |  |  |  |  |  |  |  | **0.954^**^** | **0.610^*^** | **0.968^**^** | **0.849^**^** | | **0.897^**^** | | **0.793^**^** | **0.888^**^** | | -0.087 | -0.261 | -0.258 | | | 0.590 | | -0.004 | -0.505 |
|  |  | VRBA Stage 4 |  |  |  |  |  |  |  |  |  |  |  |  |  |  |  |  |  |  |  |  |  | **0.594^*^** | **0.910^**^** | **0.879^**^** | | **0.830^**^** | | **0.814^**^** | **0.895^**^** | | -0.058 | -0.184 | -0.205 | | | **0.650^*^** | | 0.052 | -0.397 |
|  |  | VRBA Stage 5 |  |  |  |  |  |  |  |  |  |  |  |  |  |  |  |  |  |  |  |  |  |  | 0.416 | 0.279 | | **0.806^**^** | | **0.803^**^** | **0.886^**^** | | 0.097 | -0.136 | -0.138 | | | **0.804^**^** | | 0.243 | -0.265 |
|  |  | VRBA Stage 6 |  |  |  |  |  |  |  |  |  |  |  |  |  |  |  |  |  |  |  |  |  |  |  | **0.875^**^** | | **0.795^**^** | | **0.690^**^** | **0.766^**^** | | -0.082 | -0.246 | -0.237 | | | 0.448 | | -0.047 | -0.480 |
| MAS 100 | Fungi | MEA |  |  |  |  |  |  |  |  |  |  |  |  |  |  |  |  |  |  |  |  |  |  |  |  | | **0.611^**^** | | **0.465^*^** | **0.566^**^** | | -0.200 | -0.257 | -0.289 | | | 0.385 | | -0.145 | -0.529 |
|  |  | DG18 |  |  |  |  |  |  |  |  |  |  |  |  |  |  |  |  |  |  |  |  |  |  |  |  | |  | | **0.598^**^** | **0.900^**^** | | -0.077 | -0.125 | -0.236 | | | **0.741^*^** | | 0.192 | -0.426 |
|  | Bacteria | TSA |  |  |  |  |  |  |  |  |  |  |  |  |  |  |  |  |  |  |  |  |  |  |  |  | |  | |  | **0.601^**^** | | 0.161 | -0.232 | 0.042 | | | **0.839^**^** | | 0.063 | -0.390 |
|  |  | VRBA |  |  |  |  |  |  |  |  |  |  |  |  |  |  |  |  |  |  |  |  |  |  |  |  | |  | |  |  | | 0.040 | -0.191 | -0.197 | | | **0.821^**^** | | 0.165 | -0.387 |
| Button Sampler | Fungi | MEA |  |  |  |  |  |  |  |  |  |  |  |  |  |  |  |  |  |  |  |  |  |  |  |  | |  | |  |  | |  | 0.270 | **0.875^**^** | | | 0.505 | | 0.116 | -0.136 |
|  |  | DG18 |  |  |  |  |  |  |  |  |  |  |  |  |  |  |  |  |  |  |  |  |  |  |  |  | |  | |  |  | |  |  | 0.144 | | | 0.179 | | **0.869^**^** | 0.434 |
|  |  | DG18 37ºC |  |  |  |  |  |  |  |  |  |  |  |  |  |  |  |  |  |  |  |  |  |  |  |  | |  | |  |  | |  |  |  | | | 0.274 | | -0.148 | -0.174 |
|  | Azole resistance | DAS |  |  |  |  |  |  |  |  |  |  |  |  |  |  |  |  |  |  |  |  |  |  |  |  | |  | |  |  | |  |  |  | | |  | | 0.379 | -0.297 |
|  | Bacteria | TSA |  |  |  |  |  |  |  |  |  |  |  |  |  |  |  |  |  |  |  |  |  |  |  |  | |  | |  |  | |  |  |  | | |  | |  | 0.490 |
| *. Correlation is significant at the 0.05 level (2-tailed). **. Correlation is significant at the 0.01 level (2-tailed). | | | | | | | | | | | | | | | | | | | | | | | | | | | | | | | | | | | | | | | | | |

**Table S14 -** Comparison of particulate matter between summer and winter. Mann-Whitney test results.

|  | Season | N | Ranks | | Test statistics | |
| --- | --- | --- | --- | --- | --- | --- |
|  |  |  | Mean Rank | Sum of Ranks | Mann-Whitney U | p |
| Particles 0.5µ, 1.0µ, 5.0µ and 10.0µ | Summer | 19 | 19.11 | 363.00 | 173.000 | 0.347 |
|  | Winter | 22 | 22.64 | 498.00 |  |  |
|  | Total | 41 |  |  |  |  |
| Particles 0.3µ and 2.5µ | Summer | 19 | 23.05 | 438.00 | 170.000 | 0.308 |
|  | Winter | 22 | 19.23 | 423.00 |  |  |
|  | Total | 41 |  |  |  |  |


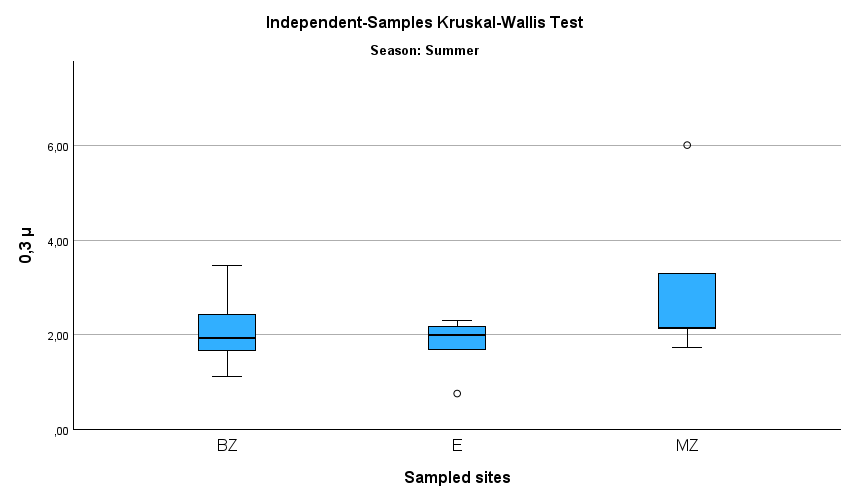


**Figure S2 -** Distribution of 0.3µ particles by sampled sites in summer


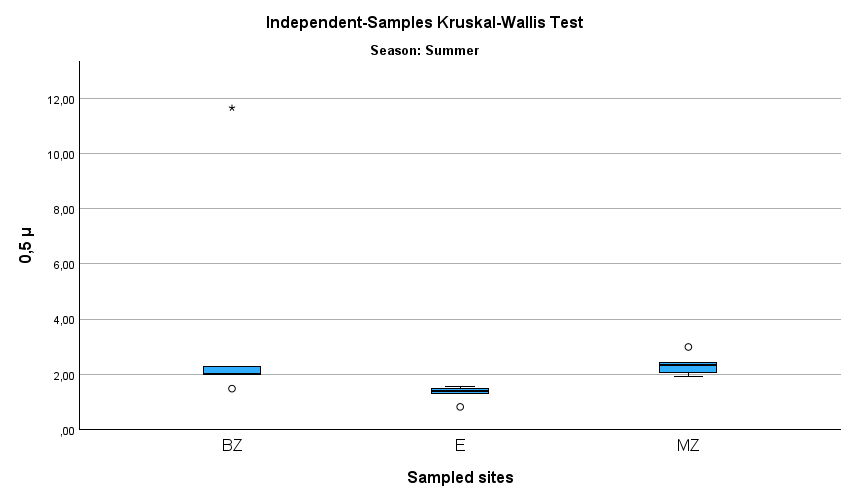


**Figure S3 -** Distribution of 0.5µ particles by sampled sites in summer


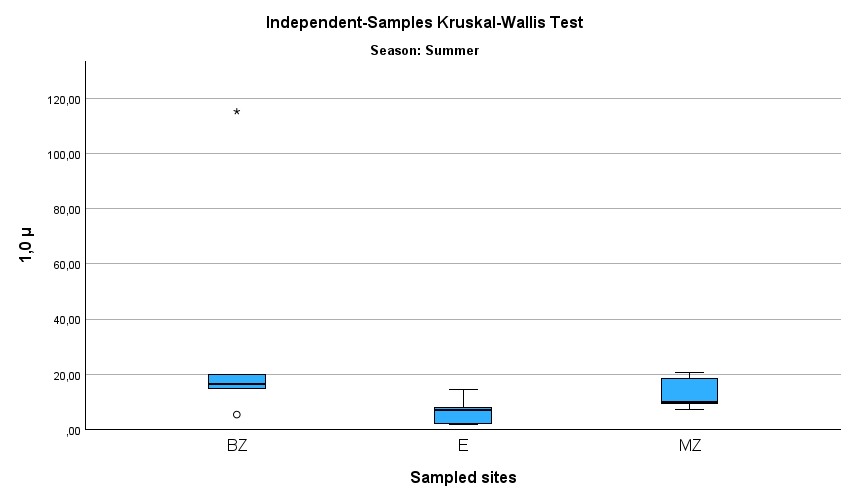


**Figure S4 -** Distribution of 1.0µ particles by sampled sites in summer

**
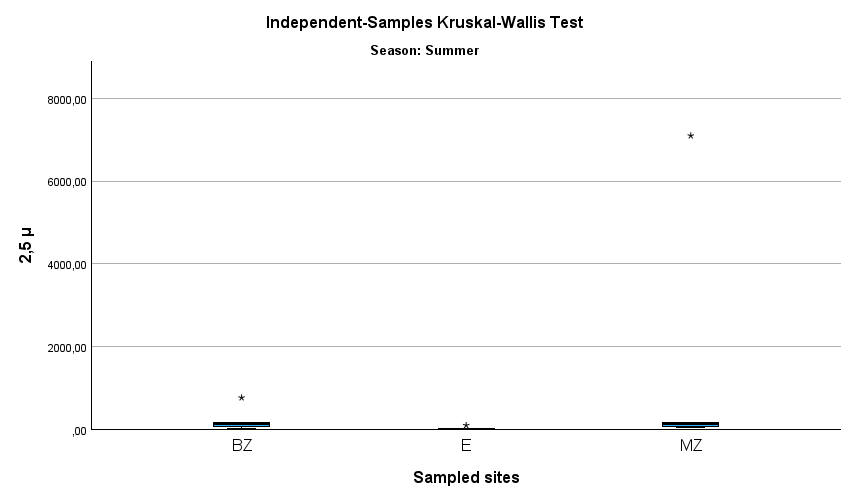
Figure S5 -** Distribution of 2.5µ particles by sampled sites in summer


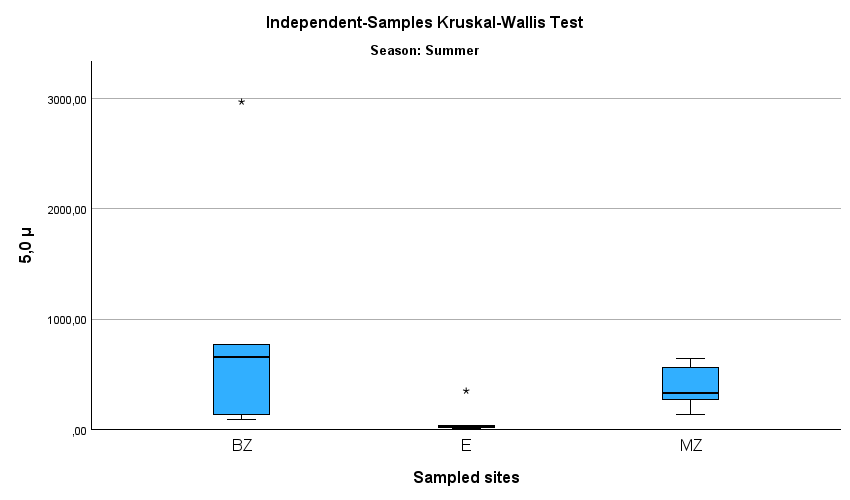


**Figure S6 -** Distribution of 5.0µ particles by sampled sites in summer


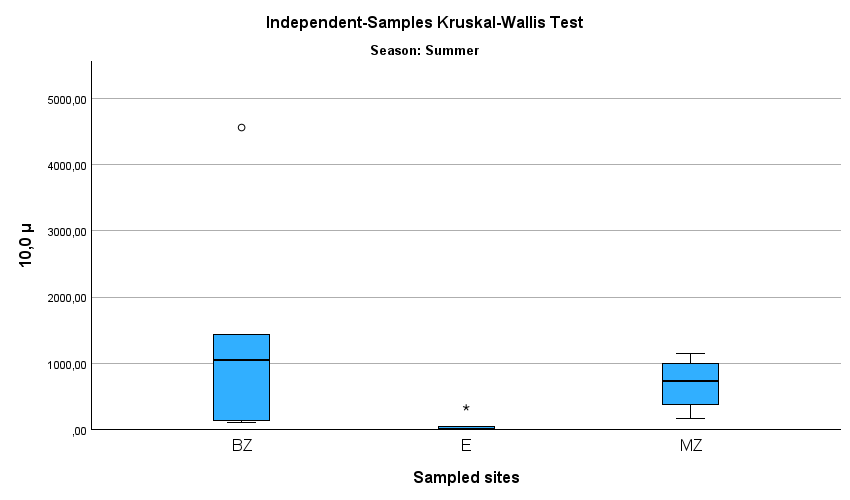


**Figure S7 -** Distribution of 10.0µ particles by sampled sites in summer

**
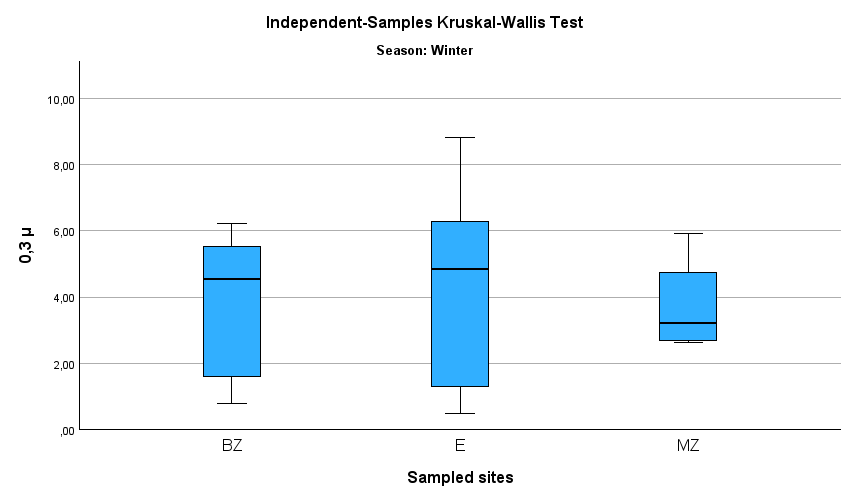
Figure S8 -** Distribution of 0.3µ particles by sampled sites in winter


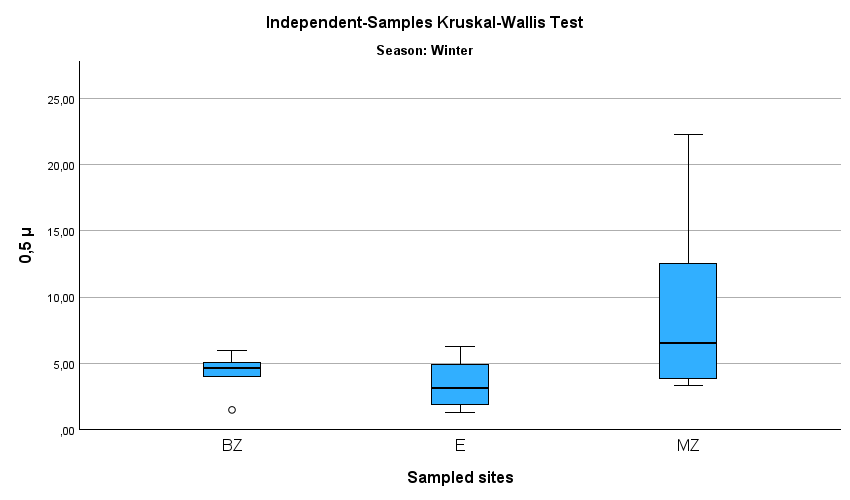


**Figure S9 -** Distribution of 0.5µ particles by sampled sites in winter


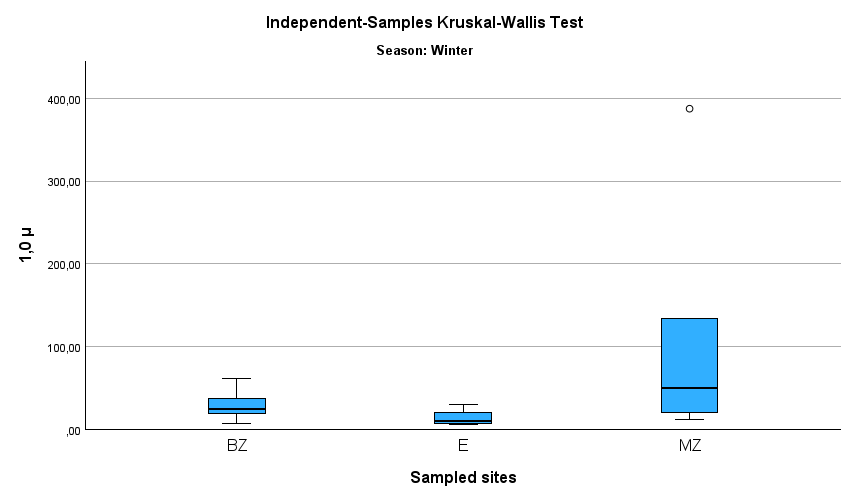


**Figure S10 -** Distribution of 1.0µ particles by sampled sites in winter

**
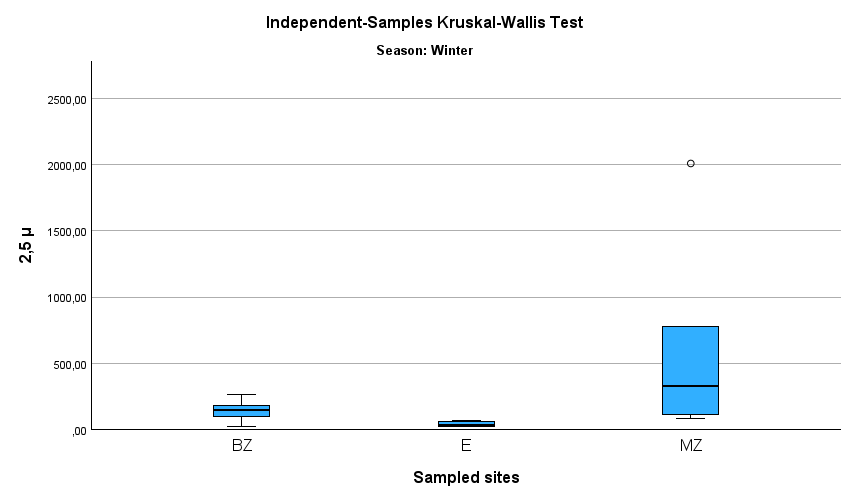
Figure S11 -** Distribution of 2.5µ particles by sampled sites in winter

**
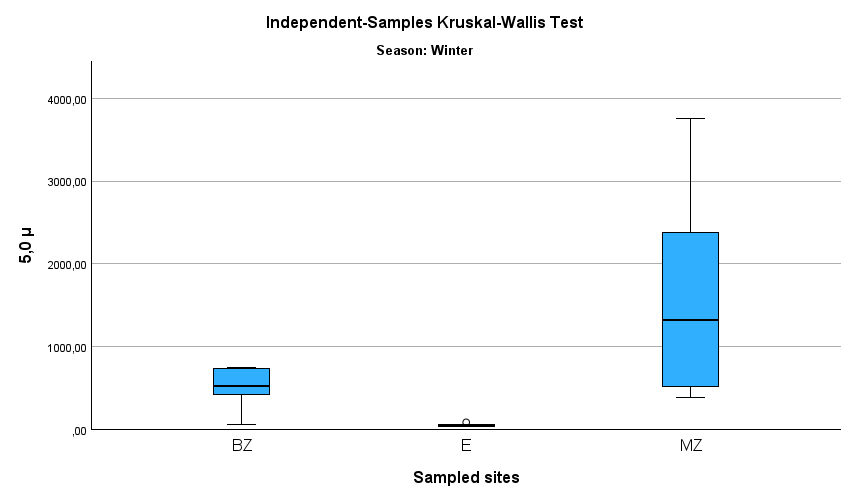
Figure S12 -** Distribution of 5.0µ particles by sampled sites in winter

**
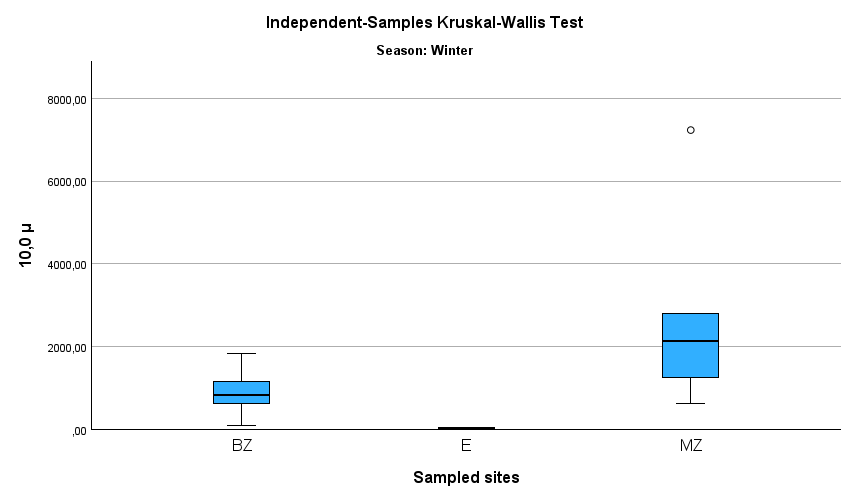
Figure S13 -** Distribution of 10.0µ particles by sampled sites in winter
